# Treatments for people who use anabolic androgenic steroids: a scoping review

**DOI:** 10.1186/s12954-019-0343-1

**Published:** 2019-12-30

**Authors:** Geoff Bates, Marie-Claire Van Hout, Joseph Tay Wee Teck, Jim McVeigh

**Affiliations:** 1 Public Health Institute, Liverpool John Moores University, Liverpool, England; 2 MRC/CSO SPHSU, University of Glasgow, Glasgow, Scotland; 3 Department of Sociology, Manchester Metropolitan University, Manchester, England

**Keywords:** Anabolic androgenic steroids, Drug treatment, Health care, Dependence, Behaviour change

## Abstract

**Background:**

A growing body of evidence suggests that anabolic androgenic steroids (AAS) are used globally by a diverse population with varying motivations. Evidence has increased greatly in recent years to support understanding of this form of substance use and the associated health harms, but there remains little evidence regarding interventions to support cessation and treat the consequences of use. In this scoping review, we identify and describe what is known about interventions that aim to support and achieve cessation of AAS, and treat and prevent associated health problems.

**Methods:**

A comprehensive search strategy was developed in four bibliographic databases, supported by an iterative citation searching process to identify eligible studies. Studies of any psychological or medical treatment interventions delivered in response to non-prescribed use of AAS or an associated harm in any setting were eligible.

**Results:**

In total, 109 eligible studies were identified, which included case reports representing a diverse range of disciplines and sources. Studies predominantly focussed on treatments for harms associated with AAS use, with scant evidence on interventions to support cessation of AAS use or responding to dependence. The types of conditions requiring treatment included psychiatric, neuroendocrine, hepatic, kidney, cardiovascular, musculoskeletal and infectious. There was limited evidence of engagement with users or delivery of psychosocial interventions as part of treatment for any condition, and of harm reduction interventions initiated alongside, or following, treatment. Findings were limited throughout by the case report study designs and limited information was provided.

**Conclusion:**

This scoping review indicates that while a range of case reports describe treatments provided to AAS users, there is scarce evidence on treating dependence, managing withdrawal, or initiating behaviour change in users in any settings. Evidence is urgently required to support the development of effective services for users and of evidence-based guidance and interventions to respond to users in a range of healthcare settings. More consistent reporting in articles of whether engagement or assessment relating to AAS was initiated, and publication within broader health- or drug-related journals, will support development of the evidence base.

## Introduction

Human enhancement drug use differs from other forms of drug use by virtue of the motivation or purpose of their use. Typically, they are not consumed either for a treatment of an illness or injury nor for instant gratification through their psychoactive properties. Instead, their function is an attempt to change an individual’s appearance or improve a skill, ability or activity [1, 2]. Characterised by man’s endeavour to gain an advantage over his competitor, their usage is by no means a new phenomenon, featured in social, ritual and sporting contexts throughout recorded history. Attempts to classify enhancement drugs have resulted in the six broad categories of drugs to increase lean muscle mass, to suppress appetite or reduce weight, to change the appearance of the hair or skin, to increase sexual desire or enhance performance, to improve cognitive function and to enhance mood or social interaction. Over the past 30 years, there has been growing media, policy and academic interest in this form of drug use, in particular the classification of drugs used to enhance musculature size and strength. Most notable within this category are the anabolic androgenic steroids (AAS) and their associated drugs [3–6]. Also included in this classification are a range of other hormones [7–11] including human growth hormone [12, 13] and insulin [7, 14].

While AAS doping remains a concern for sport, both at elite and recreational levels [15–17], the wider societal impact is now apparent [4, 18, 19]. Although prevalence estimates of clandestine behaviours such as AAS are notoriously difficult, a growing body of evidence has indicated that while well established in North America, northern Europe and Australia, there are concerns across the globe [6, 19].

In recent years, research has provided a more nuanced understanding of AAS use in relation to the diverse characteristics and motivations of users [20–27], together with knowledge of the variety and patterns of drug use from both academic studies [28–34] and other sources [35]. Extensive research and comprehensive reviews have provided details of the identified adverse health conditions experienced by users of these durgs [36], while new research has identified new and concerning health risks [37, 38] and the potential for transmission of blood-borne viruses [20, 29, 39–43].

A body of research has discussed the risk of developing AAS dependence and it is estimated that up to 30% of AAS users may develop dependence, characterised by the simultaneous use of multiple AAS in large doses over long periods of time [36, 44]. While AAS are not explicitly recognised in the Diagnostic and statistical manual of mental disorders (DSM 5) as one of nine classes of drugs [45], they may be considered under the tenth ‘other (or unknown) substance’ class. The DSM 5 determines the severity of a substance use disorder from mild to severe according to the presence of up to 11 criteria. It is argued that while there are differences between AAS and psychoactive drugs dependence, such as that AAS are typically used over a period of weeks and months to increase muscularity rather than to achieve a ‘high’ in the short-term, these criteria are still highly applicable to AAS dependence [46]. Criteria such as tolerance, withdrawal, use of the substance in larger amounts, unsuccessful attempts to reduce or stop using the substance, and time spent on activity related to the substance use have all been identified as features of AAS dependence [44, 46]. A number of hypotheses to explain AAS dependence have been put forward [47, 48] and recommendations for treating what has been described as steroid ‘abuse’ or dependence have long been proposed [49–51].

Recent recommendations to treat steroid dependence include a staged discontinuation, managing withdrawal symptoms, maintaining abstinence and attenuating complications of chronic use [51–53]. Long-term use of AAS at high doses may lead to the development of a range of withdrawal symptoms following cessation, including depression, insomnia, suicidal ideation and fatigue, which may persist for many months [47, 51, 54]. Withdrawal is characterised by psychiatric and neuroendocrine symptoms, with the user ultimately re-initiating AAS to alleviate or avoid their onset. Supporting discontinuation may require a multidisciplinary approach with input from health professionals such as a GP, addiction specialist, psychiatrist and endocrinologist [53]. Swedish guidelines for diagnosing and treating AAS ‘abuse’ [55] include advice around psychosocial treatments, such as cognitive behavioural therapy, counselling group therapy and motivational interviewing. These therapies address the user’s preoccupation with enhancing their muscularity, their experiences of past bullying or violence, and resulting self-esteem and confidence issues. Brower (2009) believes that these entrenched psychological issues should be addressed once acute withdrawal is resolved as part of successful treatment [51]. Muscle dysmorphia and associated drive for muscularity [56–58] may be risk factors for both initiating and continuing AAS use, and potentially dependence [52]. It may be necessary to identify and address such disorders through counselling or psychotherapies as part of AAS treatment to reduce likelihood of re-initiation [53].

There has been a fourfold increase in the number of English language academic papers published between 1995 and 2015 [59]. However, there remains scant evidence in relation to effective policy and practice within the topic. While we have a greater understanding of the environmental influences and risk factors for use [17, 60–62], there are few robust findings to support the effective prevention of AAS use. Little progress has been made in answering the fundamental questions of how do we make AAS less attractive and how do we make these drugs less accessible to those at risk of initiating use [63–66].

Tensions between some AAS users and the medical community are well documented [26, 67–69] and long established [70], predating anti-doping or legislative control in most countries. Although psychological harm and the potential demand for interventions to address dependence are also well recognised [71–75] and diagnostic tools available [52, 76], available services are few and far between. Harm reduction programmes, in the form of needle and syringe programmes (NSP), have clearly been successful in engaging AAS users in Australia [42, 43, 77, 78] and, in particular, the United Kingdom [5, 30, 79, 80]. However, even where uptake of service is high, substantial numbers of AAS users do not access these services [26, 68, 80, 81]. Policy guidance regarding the delivery of harm reduction services for AAS users, centred around NSP provision, is in place in the United Kingdom [82, 83], with its importance recognised in National Drug Strategy and Treatment guidelines [84, 85]. While these guidelines are based on well-established principles of treatment engagement and harm reduction, there is an urgent need to identify where we have evidence to support specific interventions and where the evidence gaps remain.

### Aims

The overall aim of this review was to identify and describe what is known about psychosocial and medical interventions that aim to support and achieve cessation of AAS, and treat and prevent associated health consequences. Specifically, the review aimed to identify:

1. What studies have examined the implementation and impact of interventions to support ASS cessation, and manage the health consequences related to cessation?
2. What studies have examined the implementation and impact of interventions to treat the harms or side effects associated with AAS use?
3. What are the implications of these findings, and what are the gaps in the evidence base that research in this area needs to address?

## Methodology

The review was undertaken following Arksey and O’Malley’s guidance for scoping reviews, which informed the development of review methods and write-up of methods and findings [86].

### Inclusion and exclusion criteria

Studies were eligible that included males or females with current or discontinued use of AAS alone, or AAS use alongside other substances. Use for any reason (for example, strength or sporting enhancement, aesthetic reasons) was acceptable with the exception of where AAS were prescribed or taken as part of a treatment regimen or in a controlled medical setting. Studies of any psychosocial or medical treatment interventions were eligible, including those that aimed to support individuals to discontinue AAS use or to treat the health consequences of current or past use. This included, but was not restricted to, treating AAS withdrawal, physical or psychological dependence, injuries, acute conditions, chronic conditions, side effects and blood-borne viruses. Studies that did not provide a description of the treatment given or those that did not describe any outcome following treatment at any follow-up time were excluded. Interventions that took place in any setting were eligible, including, but not restricted to, primary and secondary care, community settings such as drugs misuse services, NSPs and AAS clinics, sport and fitness environments, and prisons.

All types of study designs were considered due to the anticipated lack of high-quality controlled trials. Articles published in English were eligible with no date restrictions.

### Search strategy

Initially, a comprehensive search was carried out in four bibliographic databases (Medline, PsycINFO, Sports Discus and the Social Sciences Citation Index) in January 2018. A search strategy was developed initially in Medline and adapted for the other databases. The full Medline search is provided in Additional file 2.

The reference lists of all identified papers were screened to identify potentially eligible studies. Forward citation searches for included articles were executed in PubMed and the identified studies were assessed against the review inclusion criteria. This iterative process continued for all articles identified through these steps. Due to the nature of the evidence base, with studies likely to cover a broad range of topics and to be published in a wide variety of sources, these additional searches were expected to be important to identify relevant literature. Initially, titles and abstracts for all articles identified were reviewed against the inclusion criteria by one reviewer. A sample of 10% was independently reviewed by a second reviewer. The full texts for all articles included at this stage were retrieved and subjected to further screening against inclusion criteria.

### Data extraction and synthesis

The relevant characteristics of identified studies were extracted into structured tables. This included population characteristics and details of their AAS use, the symptoms requiring treatment or reasons for seeking help, diagnosis, details of the treatment given and the outcomes of this treatment. Studies were grouped by the types of harms identified in Pope and colleagues’ review of the harms associated with AAS use [36]. A formal assessment of study quality was not undertaken, as this step is not recommended for scoping reviews [86]. However, comments on the overall nature, strengths and limitations of the evidence base are provided alongside discussion of review findings.

## Results

### Identification of studies

**Fig. 1:**
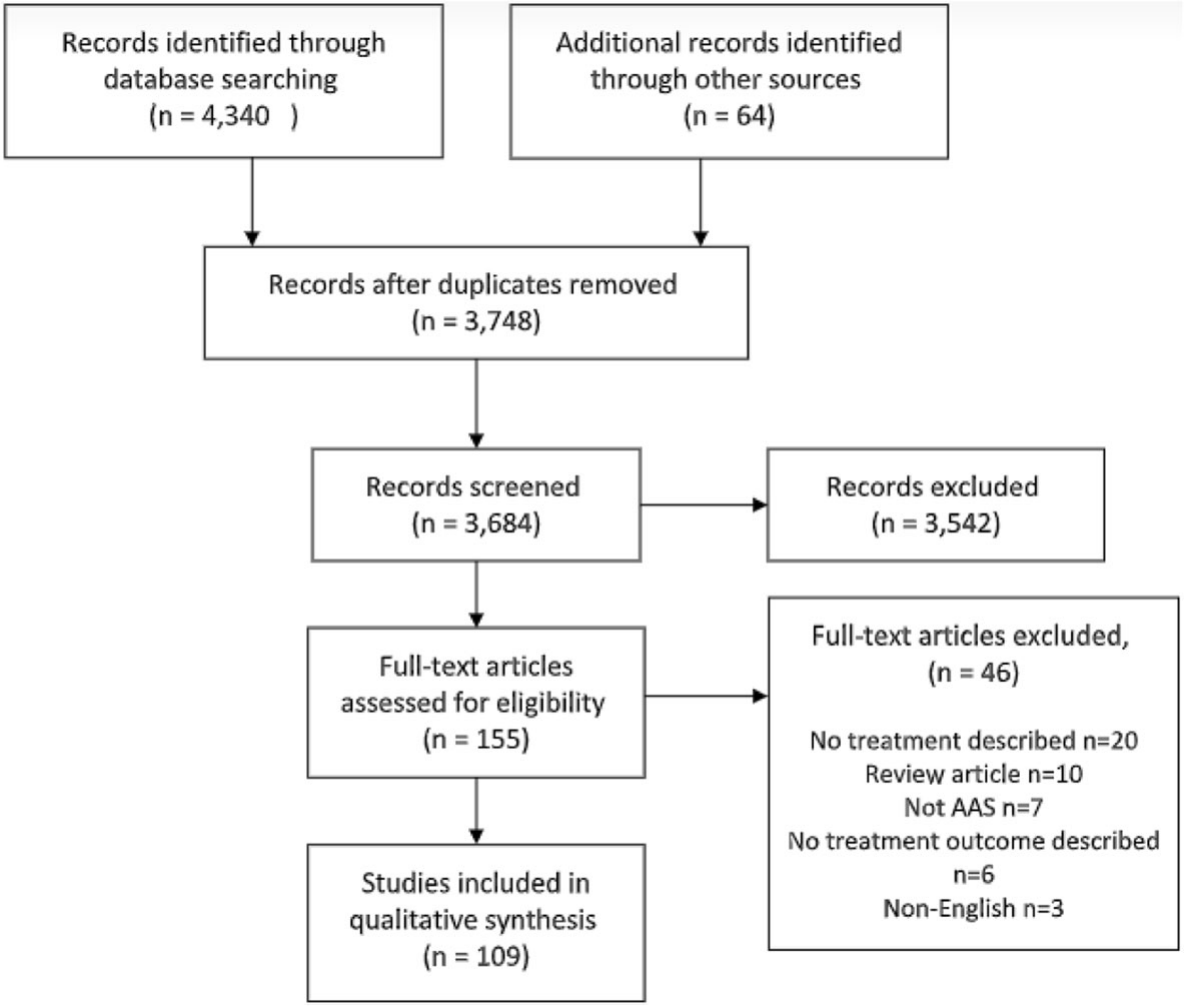
Flow of studies through the review

Database searching identified 3,684 articles. Following screening of article title and abstracts against review inclusion criteria, full-text articles were accessed for 76 articles and these were again reviewed against the inclusion criteria. An additional 64 studies were identified through checking the reference lists and citations of the included articles. These were screened in the same manner. Following full-text screening, 46 articles were excluded, predominantly because no treatments were reported. The reasons for exclusion at this stage are reported in Fig. 1.

### Summary of findings

In total, 109 studies met the review inclusion criteria. Summaries of the included studies are provided in Table 1, grouped by the type of condition that required treatment. The studies were carried out in 28 countries, most prominently the USA (*n* = 33) and the UK (*n* = 21). One study followed a retrospective chart review design with the others case report (*n* = 94) or case series (*n* = 14) designs. With the lack of any controlled studies, it was difficult to draw conclusions relating to the effectiveness of any treatments provided. Additionally, there were substantial variations across studies in the depth of reporting about participants, settings, condition requiring treatment, the treatments provided and outcomes. The identified studies were published in sources representing a diverse range of disciplines.

**Table 1:** Summary of included studies

Reference no.	Country	Study design	Diagnosis	Treatment approach
Psychiatric (*n* = 12)				
[87]	USA	CS	Depression	Medical therapy
[88]	USA	CR	Substance dependence	Drug treatment programme participation
[89]	Canada	CR	Depressed mood with anxiety, paranoia, derealisation	Medical therapy, electroconvulsive treatment
[90]	Greece	CR	Mood disorder with manic features	Medical therapy
[91]	Germany	CR	Mania	Medical therapy
[92]	USA	CR	Borderline personality disorder with antisocial traits	Medical therapy, education about AAS, psychotherapy
[93]	India	CR	Substance dependence including opioids and AAS	Medical therapy for AAS and opioid withdrawal, psycho-education and relapse prevention
[94]	Ireland	CR	Mixed psychotic disorder	Medical therapy
[95]	USA	CR	Acute mania	Medical therapy
[96]	UK	CR	Psychosis with low mood	Medical therapy
[97]	USA	CR	AAS dependence	Drug treatment programme participation
[98]	USA	CR	AAS dependence	Medical therapy
Neuroendocrine (*n* = 11)				
[99]	Netherlands	CR	Hypogonadotropic hypogonadism	Medical therapy
[100]	USA	CR	Hypogonadism	Medical therapy
[101]	Malaysia	CR	Complete azoospermia	Medical therapy
[102]	UK	CR	Severe hypogonadotropic hypogonadism	Medical therapy
[103]	UK	CS	Azoospermia	Advice to discontinue AAS
[104]	USA	CR	Impotence—reduced testicular volume on both sides and gynaecomastia on both sides	Medical therapy
[105]	USA	CR	Azoospermia	Discontinuation of all medications; medical therapy
[106]	USA	RCR	Azoospermia	AAS cessation and medical therapy
[107]	Italy	CR	Hypogonadotropic hypogonadism	Medical therapy following AAS discontinuation
[108]	USA	CS	Hypogonadism	Medical therapy
[109]	USA	CR	Azoospermia	Medical therapy
Hepatic (*n* = 25)				
[110]	USA	CR	Tumour haemorrhage in liver. On second presentation: tender hepatomegaly and haemorrhage, tachycardia	Surgery; instruction to discontinue AAS
[111]	Mexico	CR	Liver toxicity, cholestasis	Medical therapy; AAS discontinued
[112]	USA	CS	Hepatotoxicity	Medical therapy
[113]	USA	CR	Liver toxicity	Medical therapy
[114]	USA	CS	Hepatotoxicity. In one case, patient suffered from renal failure.	Medical therapy
[115]	Spain	CS	1) Hyperechogenic lesions in the liver; 2) Acute renal failure, muscular damage, metabolic alkalosis and hypernatraemia	1) Instruction to discontinue AAS; inclusion in liver transplantation program; 2) Patient received haemodialysis; instruction to discontinue AAS.
[116]	UK	CR	Hepatic rupture with cardiovascular collapse, sepsis and acute renal failure	Resuscitation, surgery
[117]	Germany	CR	Hepatocellular carcinoma. Liver was enormously enlarged	Chemoembolization was declined by patient who was recommended for transplantation
[118]	Australia	CR	Hepatocellular carcinoma	Surgery
[119]	Lebanon	CR	Liver injury resulting in prolonged cholestasis and acute kidney injury	Advice to discontinue AAS, medical therapy, plasma exchange. Patient refused renal biopsy.
[120]	China	CR	Dilated cardiomyopathy and acute hepatic injury	Medical therapy
[121]	Poland	CR	Severe intrahepatic cholestasis that developed to severe liver failure	Medical therapy
[122]	UK	CS	Cholestasis	Medical therapy
[123]	Spain	CR	Severe cholestatic jaundice	Unclear
[124]	USA	CS	Severe hepatotoxicity, cholestasis	Medical therapy
[125]	Netherlands	CR	Mild jaundice; cholestatic hepatitis identified through liver biopsy	Medical therapy
[126]	UK	CR	Three grade II oesophageal varices	Blood transfusion and sclerotherapy
[127]	Australia	CR	Intrahepatic cholestasis	Medical therapy
[128]	USA	CR	Severe cholestasis and renal failure. Re-admitted with pruritus	Medical therapy
[129]	Spain	CR	Hepatic rupture, liver failure. Hematoma of the liver	Surgery
[130]	USA	CR	Acute, nonobstructive, intrahepatic cholestatic hepatitis	Medical therapy; advice to avoid other medications
[131]	USA	CR	Severe jaundice, bile acid nephropathy	Medical therapy, blood transfusion, AAS discontinued
[132]	Spain	CS	Severe cholestasis, hepatotoxicity	Medical therapy followed by MARS therapy
[133]	Brazil	CR	Giant hepatic adenoma	Surgery
[134]	Germany	CR	Hepatocellular carcinoma	Surgery
Kidney (*n* = 6)				
[135]	USA	CR	Initial diagnosis of hepatic adenomatosis (2004). On third admission, diagnosed with chronic kidney disease and coronary artery disease (2013)	Advice given to discontinue AAS initially. Surgery at later presentation.
[136]	Spain	CR	Severe acute kidney failure with high blood pressure, anaemia and thrombocytopenia	Medical therapy
[137]	Iran	CR	Acute renal failure; muscle injury and rhabdomyolysis	Medical therapy
[138]	Brazil	CS	Acute kidney injury in both cases	Medical therapy.
[139]	USA	CR	Recurrent renal infarction	Medical therapy, AAS counselling
[140]	Lebanon	CR	Acute pancreatitis, acute renal failure and hypercalcemia.	Medical therapy
Cardiovascular (*n* = 26)				
[141]	Japan	CR	Cardioembolic stroke	Medical therapy, AAS use discontinued
[142]	Sweden	CR	Intraparenchymal haemorrhage in right parietal lobe; right cortical venous thrombosis	Anticoagulation therapy
[143]	UK	CR	Acute myocardial infarction	Surgery
[144]	Egypt	CR	Severe toxic cardiomyopathy.	Medical therapy
[145]	Canada	CR	Cardiomyopathy	Incubation, medical therapy, resuscitation, dialysis and device implantation, addiction counselling referral
[146]	Argentina	CR	Posterior territory ischemic stroke.	Intubation and ventilation; rehabilitation
[147]	Sweden	CR	Severe hypertension	Aggressive treatment with intravenous drugs; AAS cessation
[148]	Turkey	CR	Acute coronary syndrome	Medical therapy
[149]	Sweden	CS	i) Occlusion of all major arteries of the leg. ii) Arterial thrombosis:	i) Surgery ii) Thrombolysis attempted with no improvement. Surgery performed.
[150]	Canada	CR	Stroke. Upon readmission 3 years later, diffused distal arterial thrombosis	Medical therapy
[151]	Kuwait	CR	Cardiomyopathy, stroke and peripheral vascular disease	Medical therapy
[152]	Portugal	CR	Severe toxic cardiomyopathy.	Medical therapy
[153]	USA	CR	Myocardial infarction	Medical therapy
[154]	Turkey	CR	Myocardial infarction	Medical therapy
[155]	Greece	CR	Myocardial infarction	Medical therapy
[156]	USA	CR	Acute myocardial infarction and polycythaemia	Surgery, medical therapy, phlebotomy.
[157]	Portugal	CR	Myocardial infarction	Medical therapy.
[158]	Turkey	CR	Acute inferior myocardial infarction, renal infarction	Medical therapy, surgery
[159]	USA	CR	Acute myocardial infarction	Medical therapy, rehabilitation
[160]	USA	CR	Myocardial infarction	Medical therapy
[161]	Australia	CR	Persistent atrial fibrillation	Electrical cardioversion, medical therapy.
[162]	Germany	CR	Severe coronary heart disease	Surgery, medical therapy
[163]	UK	CR	Coronary thrombus	Medical therapy
[164]	USA	CR	Cardiomyopathy, severe systolic dysfunction and Class IV heart failure.	Medical therapy and device implementation until discharge.
[165]	USA	CR	Cardiomyopathy, acute systolic heart failure.	Medical therapy, instruction not to use AAS.
[166]	Finland	CS	Cardiac hypertrophy	Surgery, medical therapy. In one case, no treatment was reported
Musculoskeletal (*n* = 13)				
[167]	USA	CR	Tear in the midsubstance of the triceps tendon.	Surgery, immobilisation
[168]	Israel	CR	Massive rhabdomyolysis	Medical therapy
[169]	Ireland	CR	Quadriceps tendon rupture, patella tendon rupture, distal femur fracture, patella dislocation in both legs	Surgery, immobilisation, physiotherapy
[170]	Iran	CR	Quadriceps tendon rupture in both knees and partial rupture of triceps tendon.	Surgery, immobilisation, physiotherapy
[171]	UK	CR	Bilateral rupture of the quadriceps tendon	Surgery, immobilisation
[172]	Denmark	CR	Complete rupture of the extensor pollicis longus tendon.	Surgery, immobilisation
[173]	Finland	CR	Complete bilateral quadriceps tendon rupture in both legs	Surgery, immobilisation
[174]	UK	CR	Rupture of both quadriceps tendons	Surgery, immobilisation, physiotherapy
[175]	Finland	CR	Bilateral distal biceps tendon avulsions	Surgery, immobilisation, physiotherapy
[176]	UK	CR	Complete rupture of the anterior cruciate ligament	Physiotherapy
[177]	UK	CR	Rhabdomyolysis. Initially diagnosed with musculoskeletal pain.	Medical therapy
[178]	UK	CR	Bilateral simultaneous traumatic upper arm compartment syndromes	Surgery
[179]	Italy	CR	Complete tear of quadriceps tendon	Surgery, immobilisation, rehabilitation
Infectious (*n* = 7)				
[180]	USA	CR	Abscess.	Medical therapy; AAS counselling
[181]	Israel	CR	Full thickness skin and subcutaneous tissue necrosis	Surgery
[182]	USA	CR	Pyomyositis	Medical therapy, surgery
[183]	UK	CS	Injection injury	Surgery, medical therapy
[184]	Turkey	CR	Spontaneous corpus cavernosum abscess	Surgery
[185]	UK	CR	Necrotizing myositis	Surgery, medical therapy
[186]	UK	CR	Abscess	Surgery, medical therapy
Other (*n* = 8)				
[187]	UK	CR	Chronic laryngitis	Medical therapy followed by laser treatments
[188]	UK	CR	Hypokalaemia and metabolic alkalosis.	Fluid provision
[189]	UK	CR	Abnormal lipid profile	Advice to stop using AAS
[190]	UK	CR	Acute respiratory distress syndrome	Intubation and ventilation; rehabilitation.
[191]	USA	CR	Multiple organ dysfunction syndrome, acute kidney injury and refractory supraventricular tachycardia	Resuscitation, medical therapy, ventilation, haemodialysis and electrical cardioversion for different symptoms.
[192]	USA	CR	New onset of diabetes	Medical therapy, AAS advice
[193]	Lebanon	CS	Spontaneous subdural haematoma	Surgery
[194]	UK	CR	Bilateral internal laryngocoeles	Medical therapy

*CR* case report, *CS* case series, *RCR* retrospective chart review

Across the included studies, all participants were male. They included a wide range of ages, with the majority in their 20s and 30s, and represented a broad range of experience using AAS from recent initiators to long-term use. Participants’ motivations and history were not reported in a consistent manner to understand factors driving AAS use, but they were frequently described as participating in bodybuilding or weight-lifting activities. The types of conditions requiring treatment included psychiatric (*n* = 12), neuroendocrine (*n* = 11), hepatic (*n* = 25), kidney (*n* = 6), cardiovascular (*n* = 26), musculoskeletal (*n* = 13) and infectious (*n* = 7). A further eight studies were categorised as ‘other’ disorders. In a small number of studies, participants were diagnosed with multiple conditions, but they have been grouped by the primary diagnosis.

Further details on participants’ AAS use, conditions requiring treatment, the treatments provided and outcomes are provided in Additional file 1.

### Treatment to support AAS cessation

Four studies reported abstinence-focussed interventions following a diagnosis of AAS dependence. In two cases, patients participated briefly in a drug treatment programme [88, 97] before withdrawing. In one, the patient received medication and psychosocial interventions to manage AAS and opioid withdrawal [93] and withdrawal symptoms abated over time. Detail on the nature of these treatments was not provided. In the remaining study, the patient received medication for a short period before deciding to resume their AAS use due to withdrawal symptoms [98]. There was no evidence identified here, however, regarding psychosocial interventions that have sought to address any associated psychological disorders amongst users seeking treatment for their AAS use or any other condition. Additionally, no evidence was identified on approaches to reduce risk of relapse by developing social support systems, improving self-confidence or managing stress, all identified as potentially important factors to be addressed during AAS treatment [51, 52, 55].

Two studies were identified in this review where individuals who discontinued AAS use needed treatment for subsequent psychiatric symptoms including depression and suicidal ideation [87, 89]. A further 11 studies reported treatments for neuroendocrine disorders, primarily with men who had discontinued their AAS use prior to the onset of symptoms. Administering AAS suppresses the hypothalamic–pituitary testicular axis, particularly when used in large amounts and for long periods, and inhibits production of testosterone [195]. Men who discontinue long-term AAS use are at risk of hypogonadism and while this may frequently be temporary and resolve spontaneously, it may in some cases persist for long periods after cessation, requiring medical treatment [51, 196–198]. Symptoms of hypogonadism may be behind the withdrawal experiences of people with a dependence on AAS [51]. These difficult experiences have been identified as an influencing factor in users’ decisions to continue or re-instate AAS use [52]. The limited evidence here shows that positive outcomes are consistently reported in the treatment of men suffering with neuroendocrine disorders following AAS cessation.

#### Treatment for harms associated with AAS use

The bulk of the evidence identified related to current or former users receiving treatment for an acute or chronic condition or injury associated with their AAS use. This included psychiatric disorders (*n* = 12), hepatic and kidney disorders (*n* = 31), cardiovascular disorders (*n* = 26), musculoskeletal disorders (*n* = 13) and a range of other disorders (*n* = 8). The management of such conditions in the AAS-using group is similar to that of the general population [53] and details are described in the tables in the additional material provided. There was, however, limited evidence of engagement with users regarding their AAS use as part of their more general treatment. There were examples where participants were stated to have discontinued AAS following treatment and remained abstinent at follow-up [133, 157, 159], but patients’ AAS status at this time was not routinely reported.

#### Treatment as an opportunity for engagement

In a small proportion of studies (*n* = 10), it was reported that some form of intervention to bring about, or maintain change in AAS use was included as part of the treatment provided. This was most commonly instruction or advice to discontinue AAS use, with a more substantial element such as counselling only reported in three studies [139, 145, 180]. Where reported, such efforts were based on suppling risk information associated with AAS but not support with discontinuation, such as managing withdrawal symptoms. No form of harm reduction interventions were initiated alongside or following any treatments provided. Only one study [145] reported signposting or referral to another service for further support.

In comparison to people who use other psychoactive drugs, AAS users are less likely to suffer acute adverse effects from their substance use, or to have their occupational performance or relationships impaired and are, therefore, less reliant upon health professionals [44]. Research has consistently indicated this group to be reluctant to seek medical help or engage with health professionals [67, 199–201]. Where health professionals identify AAS use in a patient and are providing treatment for an associated harm, this may, therefore, provide a rare opportunity to motivate changes in behaviour. There were examples in this review of studies that included recent initiators. For example, in 12/25 studies included here reporting hepatic disorders, patients had initiated AAS use fewer than 6 months prior to treatment. Contact with a health professional at this stage could provide a valuable opportunity to engage with the individual about their motivations and substance use before habitual use develops or becomes entrenched, or identify and treat any underlying factors. In a further 5/25 studies, long-term AAS use of over 5 years was reported, and up to 15 years. For such individuals, this contact could provide opportunity to test for disorders associated with long-term use, promote behaviour change and discuss long-term plans for discontinuation of use.

#### Encouraging discontinuation and delivering harm reduction with patients treated for a disorder associated with AAS

Where a patient is receiving treatment, there will be a range of factors that affect the appropriateness of delivering any form of AAS intervention or investigating any other potential harms. For example, in many of the studies identified, the individuals treated had discontinued their AAS use a substantial time prior to seeking treatment. Additionally, many were diagnosed with acute conditions, for which immediate, and in some cases substantial, treatment was required. In such cases, it is not surprising that the acute harm will be the focus of the treatment. However, where AAS use is suspected or confirmed, a number of diagnostic tests may be appropriate to identify potential physiological or psychiatric harms [53]. Recommendations for general practitioners who identify AAS use in a patient include strongly encouraging cessation and management of withdrawal symptoms in those that do discontinue, as well as information on injecting practices, promoting alternatives to AAS and informing about long-term health harms for those who continue to use [202]. Continued encouragement and monitoring of psychiatric and physiological complications is recommended for those who are not prepared to consider discontinuation [53].

An instruction not to use AAS may be effective in some cases, but for individuals who are highly motivated to use AAS in response to a desire to change their appearance or performance, it may have little impact. Experiencing harm or increasing knowledge of potential risks may not only reduce motivation to use amongst users who may accept risks as a potential consequence of use, but also one that they can manage through their practices [60]. Where it is identified that users intend to continue administering AAS following treatment, it is important that they receive appropriate harm reduction advice, such as on safe injecting, blood-borne viruses (BBVs) and AAS cycles. For example, in seven studies, treatments for infectious complications associating with injecting AAS were reported. There was no indication of relevant harm reduction work included alongside treatment, such as advice or demonstration relating to injecting or injecting techniques in any of these studies, with the exception of Rich and colleagues who reported provision of counselling on the risks of BBVs [180].

## Discussion

Research over the past 30 years has provided a far richer understanding of the populations of AAS users, their characteristics, behaviours and motivations. While the specific risks attached to each AAS and the probability or magnitude of harm associated with highly individualised and complex drug regimens cannot be known, we now have a far greater understanding of the potential harms caused by these drugs. However, the evidence base for interventions has not kept pace. The examples of treatment identified in this review were set within primary and secondary care facilities. No studies were identified that explored the effectiveness of any approaches to encourage cessation or treat dependence within other settings where health professionals are likely to encounter users, such as steroid clinics, drugs services or NSPs. Consequently, there is a lack of any evidence on the effectiveness of such services for bringing about behaviour change in users. Within any setting there is scarce evidence on treating AAS dependence, including initiating and maintain cessation and managing withdrawal symptoms outside of case reports of former users seeking support for neuroendocrine disorders.

The findings of this scoping review are characterised by missed opportunities. While the failure to report good practice or supplementary activity is not proof that it does not occur, without confirmation we cannot make assumptions. The extensive literature outlining the symptomatic treatment of AAS-related harms within numerous medical and surgical specialisms fails to provide evidence of intervention or referral to address the major causative factor, the patients’ AAS use. This scoping review has reported only a sample of the myriad of case reports involving the treatment of AAS-related harms. These case reports not only demonstrate the lack of evidence of intervention effectiveness to support the cessation of AAS use or reduce the associated harms, they also fail to show that actual activity occurred. As a minimum, future case reports should report if any assessment for AAS dependence were conducted. Details of advice or interventions provided to AAS users or any referral or signposting are also essential information. Referrals to primary care, endocrinologists, addiction specialists or harm reduction providers are essential building blocks in identifying care pathways and potential effective interventions. Case reports are published predominantly in clinical journals, often relating to medical or surgical specialisms. The publication of reports in broader health or public health journals or journals related to drug use, addiction or harm reduction would facilitate the inclusion of clinical experiences within a wider approach to addressing the harms associated with AAS use.

Despite the comprehensive research and literature relating to AAS dependence, there remains little evidence regarding effective interventions to support cessation of use or management of withdrawal. It is hoped that the development diagnostic tools [46], guidelines for clinical management [85] and harm reduction [82] or the commissioning of health services [83] will be accompanied by robust research and evaluation. Evaluations to date have been small scale and lack generalizability.

In addition to the need to ensure accurate and consistent reporting of activity and an upscaling of research and evaluation, there is a need to ensure that interventions are culturally appropriate to the target groups. Much of the work to date has focused on the bodybuilding communities of North America, Northern Europe and Australia. It is clear that AAS use is a global issue, with research emerging from low–middle income countries around the world in addition to industrialised high-income states. Of added significance is the diversity of individual AAS users. Interventions will need to be tailored to meet the varied characteristics and motivations of users, going beyond those looking to achieve a stylised “bodybuilding appearance” or excel at sport or even the young males attempting to bulk up. Evidence from the United Kingdom indicates that there are as many AAS users over 40 years of age as there are those under the age of 25 years [31]. It is well established that AAS use is not restricted to men and while rates amongst women are much lower [203], the complexities of treatment and care are undoubtedly much higher [23, 204, 205]. Prevalence of AAS use is higher amongst groups with specific characteristics such as professions where size or strength is an asset [206–209], amongst gay and bisexual men [20, 22, 29, 210, 211] and those using or who have previously used other drugs [212] [30, 33, 67, 212–214]. These “sub groups” may or may not require specific interventions and may merely illustrate the complexities of human nature. The majority of AAS users will not initiate or continue AAS by virtue of membership of one of these groups but will have a range of susceptibilities and motivations for use.

Beyond these challenges, to develop effective services for users of AAS is the ongoing lack of confidence that some communities of AAS users feel towards health care professionals and primary care in particular [30, 67, 199] and a feeling that reliable and relevant health information can be gained elsewhere [215]. Built on the long-standing dismissive approach towards the effectiveness of anabolic steroids by elements of the health profession [216, 217] and an ongoing ‘just say no’ stance amongst some practitioners, it is evident that establishing trust through listening to the AAS-using communities will be an essential element of intervention and service development [26].

## Conclusions

This scoping review of the literature has identified treatments given to AAS users for a wide range of physiological and psychological harms. Despite the large number of articles identified, the evidence base consists of case reports of predominantly treatment of physiological harms and there is scarce evidence on treating dependence, managing withdrawal, or initiating behaviour change in users in any settings. Evidence is urgently required to support the development of effective services for users and of evidence-based guidance and interventions to respond to users in a range of healthcare settings. More consistent reporting in articles of whether engagement or assessment relating to AAS was initiated, and publication within broader health- or drug-related journals, will support development of the evidence base.

## Supplementary information

**Additional file 1.** Data extraction tables. The data extraction tables contain the full data extracted from the 109 articles included in the review. This includes participant information, condition requiring treatment, the treatment provided and the outcomes of treatment.

**Additional file 2.** Search strategy. The full search strategy used in Medline is provided.

## Data Availability

All data generated or analysed during this study are included in this published article and its supplementary information files.

## Abbreviations

AAS: Anabolic androgenic steroids
BBV: Blood-borne virus
DSM: Diagnostic and statistical manual of mental disorders
NSP: Needle and syringe programme
