# Supplementary material for: Treatments for people who use anabolic androgenic steroids: a scoping review
Source: Harm Reduct J. 2019 Dec 30;16:75. doi: 10.1186/s12954-019-0343-1 (PMC6937954; doi:10.1186/s12954-019-0343-1)
Supplement: Supplementary file 1 — Additional file 1. Data extraction tables. The data extraction tables contain the full data extracted from the 109 articles included in the review. This includes participant information, condition requiring treatment, the treatment provided and the outcomes of treatment. [file 12954_2019_343_MOESM1_ESM.docx]

**Data extraction tables**

Extracted data for all articles included within the review (n=109) is presented by condition. Firstly, a summary of the participant(s) and condition is presented, followed by details of the treatment provided and treatment outcomes.

**Table 1. Psychiatric (n=12)**

1. **Summary of condition**

| **#** | **Citation** | **Study design (country)** | **Patient(s) description** | **Patient(s) AAS status** | **Presentation setting and symptoms** | **Condition requiring treatment** |
| --- | --- | --- | --- | --- | --- | --- |
| 24 | Malone and Dimeff, 1992 (USA) | Case series | 4 patients aged 27-31, males. | All patients had history of AAS use of between 2 and 8 years. All had discontinued up to 14 months prior to presenting. | All patients reported symptoms of depression when not taking AAS/ since discontinuing AAS as well as other symptoms including decreased energy and appetite. Three patients reported suicidal ideation, one reported frequent panic attacks. | All patients diagnosed with depression. |
| 31 | Hays et al., 1990 (USA) | Case report | 22 year old male. | 9 month history of AAS use with no break for more than 2 weeks at any time in past 6 months. Use included oxandrolone (25mmg/day), nandrolone phenpropionate, testosterone cypionate, methandrostenolone (details not reported) | Presented to chemical dependency unit stating he could not stop using AAS and was depressed, sleeping less, lacked energy, irritable and had occasional temper outbursts. Experienced craving AAS and decreased self-esteem regarding muscularity. | Substance dependence |
| 54 | Allnutt and Chaimowitz, 1994 (Canada) | Case report | 20 year old, male  Bodybuilder (not clear if competing) | 2 year history of AAS use up to 2 months prior to presenting. Used testosterone cypionate (200mg x 3 days) and testosterone proprionate (100mg x 3 days). | Patient presented at psychiatric emergency room with depression and suicidal ideation that worsened since AAS cessation. | Depressed mood. Identified as having “slightly depressed” testosterone level and mild testicular atrophy. Later developed symptoms of anxiety, derealisation, paranoia. |
| 55 | Papazisis, 2007 (Greece) | Case report | 25 year old male  History of psychotic depression | AAS use since early adolescence. Increased dose (>2g/ week) one year before admission and started using other IPEDs including growth hormone, somatrotropin, oestrogen antagonists, stimulant amino acids. | Involuntarily admitted to psychiatric hospital following being injured in a fight. He displayed psychomotor agitation, restlessness, verbal aggression, irritability and euphoria. Speech was severely disturbed. | Mood disorder with manic features. |
| 59 | Gahr et al., 2012 (Germany) | Case report | 38 year old male  Bodybuilder | Used mesterolone (25mg/day) for 3 weeks previous to admission. | Admitted to psychiatric ward with disturbed contact behaviour, logorrhea, incoherent thinking, reduced attention and appetite, increase of impulse, euphoric mood and reduced need to sleep | Mania |
| 61 | Rashid et al., 2000 (USA) | Case report | 40 year old male  25 year history of substance use including cannabis, LSD, cocaine and stimulants. | History of intermittent AAS use.  In 3 months prior to admission had used testosterone (200mg x4/day). | Previously referred to psychiatric outpatient service with chronic feelings of depression since age 16, periods of elation alternating with irritability and generalised anger, occasional insomnia. | Borderline personality disorder with antisocial traits; low testosterone |
| 65 | Ranjan et al., 2014 (India) | Case report | 28 year old male | 4 year history of prescribed AAS use. Prescribed nandrolone by medical practitioner to gain strength following opioid withdrawal. | Patient presented to drug treatment centre with AAS dependence, symptoms included hair loss and increased aggression | Substance dependence including tobacco, opioids and steroids. |
| 150 | Duffy & Kelly, 2015 (Ireland) | Case report | 19 year old male suffering from PTSD. 2 year history of cannabis use (up to 3oz. weekly) | Used methandrostenolone for 3 weeks. | First presented to emergency department with pain all over his body. Presented three times more with pain and discomfort. | Initially diagnosed with rhabdomyolysis, but noted to be hypomanic in hospital. On 4^th^ presentation, he was diagnosed with mixed psychotic disorder. |
| 151 | Franey & Espiridion, 2018 (USA) | Case report | 30 year old male with cannabis use and history of anxiety, depression, PTSD and opioid addiction. | Reported by family and friends to use AAS recently. Patient did not provide details. | Brought by police to emergency department following 3 week history of volatile behaviour | Acute mania |
| 157 | Stanley & Ward, 1994 (UK) | Case report | 27 year old male  Bodybuilder | AAS user (no details provided) | Following arrest for violent offences, symptoms included delusions of control and auditory hallucinations | Psychosis, with low mood after cessation of AAS |
| 183 | Brower et al., 1989 (USA) | Case report | 24 year old male  Non-competitive weight lifter | Used AAS for 1 year including continuously for past 9 months. At time of presentation he was using testosterone cypionate (200mg), nandrolone decanoate (100mg) every 3 days; oxandrolone (25mg), methandrostenolone (40mg) every day; bolasterone (30-45mg) & HCG (1000-2000 units) every 2-3 days | Requested help to discontinue AAS use as felt unable to stop. Presented with depression, outbursts of anger, suicidal thoughts. | AAS dependence |
| 184 | Tennant et al., 1988 (USA) | Case report | 23 year old male  Bodybuilder | Used AAS for 3 years, including during the previous 2 months: methandrostenolone (75mg AOD), methenolone (150mg AOD), oxandrolone (20mg/day), oxymetholone (100mg/day). | Reported being addicted to AAS and could not stop taking them without experiencing withdrawal symptoms | AAS dependence |

1. **Treatment and outcomes**

| **#** | **Citation** | **Treatment provider and setting** | **Summary of treatment approach** | **Detail of medication(s) or therapy provided** | **Treatment outcomes** | **Comments** |
| --- | --- | --- | --- | --- | --- | --- |
| 24 | Malone and Dimeff, 1992 (USA) | Outpatients | Medical therapy | All patients received 20mg/day fluoxetine. One patients received lorazepam (1mg t.i.d) for one month, then tapered off, for anxiety. | All patients improved within 6 weeks of fluoxetine administration. Depression symptoms were absent at follow up times of 2 months to 2 years. In one patient, anxiety symptoms improved. |  |
| 31 | Hays et al., 1990 (USA) | Inpatient | Drug treatment program | 1 week participation in chemical dependency unit program | Patient withdraw from programme after 1 week due to lack of insurance coverage. Author states that patient’s mood had improved. | Treatment not described and only brief |
| 54 | Allnutt and Chaimowitz, 1994 (Canada) | Initially: treated by endocrinologist as an outpatient  Later: hospital inpatient | Medical therapy as outpatient continued as inpatient after re-admission with electroconvulsive treatment | Prescried desiparmine (250mg/day) for 2 months. Following re-admission, patient initially given a combination of desiparmine, haldol & lithium for 7 weeks, followed by Prozac for 8 weeks and haldol recommenced for 12 weeks. After total 19 weeks the patient received 7 electroconvulsive treatments. | Improvement in depressive symptomatology following electroconvulsive treatments; discharged on desiprimine. |  |
| 55 | Papazisis, 2007 (Greece) | Inpatient | Medical therapy | Received haloperidol (20mg/day) and chlorpromazine (300mg/day) | Treatment incomplete. Following a later car accident, patient received further treatment and stopped using AAS. Patient committed suicide six months later. |  |
| 59 | Gahr et al., 2012 (Germany) | Inpatient | Medical therapy | Initially treated with olanzapine that was gradually increased up to 30mg/day. Discontinued due to increase in liver enzymes and amisulpride administered at 800mg/day. Patient developed parkinsonism and antimanic effect insufficient, so amisulpride reduced (400mg/day) and lithum carbonate administered (1800mg/day). | Within 2 months the patient developed complete remission and was without psychopathological findings at 6 month follow up. |  |
| 61 | Rashid et al., 2000 (USA) |  | Medical therapy initially, later more medical therapy, AAS discontinued, education, psychotherapy, consultation with endocrinologist | Medication with slow release lithium carbonate (1800mg/day), Prozac (80mg/day) for psychiatric symptoms. Later, Prozac decreased to 20mg/day gradually and lithium replaced with valproic acid (1800mg/day). Patient made a contract to stop taking testosterone at this point and was provided with AAS education leaflets and psychotherapy regarding his AAS use and self-image. | Improvements over time in patient’s behaviour and suicidal ideation; patient no longer desired to use AAS and reported feeling positive about the future. |  |
| 65 | Ranjan et al., 2014 (India) | Not reported | AAS discontinued, medical therapy for AAS and opioid withdrawal; psychoeducation; relapse prevention | Opioid withdrawal managed with buprenorphine and dependence treated with antagonist therapy. Symptomatic management of AAS withdrawal symptoms including fatigue, depressed mood, anxiety and decreased libido. Provided with psychoeducation and relapse prevention sessions regularly. | Symptoms of withdrawal including fatigue, weakness, depression, anxiety, insomnia and decreased libido persisted initially but LH and testosterone levels returned to normal after three months | AAS treatment is not provided in detail, only an outline |
| 150 | Duffy & Kelly, 2015 (Ireland) | Inpatient in acute admissions unit | Medical therapy | Treated with olanzapine (10mg/2x daily), occasional haloperidol and gradually reducing doses of diazepam. | Psychotic symptoms and homicidal ideation resolved over 2 months and patient was discharged on olanzapine (10mg/day). At 9 month follow up he was not experiencing any psychiatric symptoms and was not using AAS | Patient was using large amounts of cannabis and continued to do so following treatment. |
| 151 | Franey & Espiridion, 2018 (USA) | Inpatient | Medical therapy | Intially started on olanzapine (10mg PO QD) with ziprasidone (20mg IM) and Iorazepam (2mg IM) provided in response to violent episodes. In week 2 olanzapine was titrated (15mg QD) and chlorpromazine was added (100mg PO QID) due to no improvement. | Mania and psychosis improved and patient was discharged on chlorpromazine (100mg PO TID) and olanzapine (15mg PO QD). States that patient had not been readmitted to any psychiatric unit since. |  |
| 157 | Stanley & Ward, 1994 (UK) | Unclear | Medical therapy | Treated with intramuscular zuclopenthixol | Psychotic symptoms improved (does not report treatment or outcomes relating to low mood) |  |
| 183 | Brower et al., 1989 (USA) | Inpatient | Drug treatment program | Participated in chemical dependency program for 4 days | Left program on day 5, reason unknown |  |
| 184 | Tennant et al., 1988 (USA) | Outpatient | Medical therapy | Treated with clonidine for six days | Withdrawal symptoms appeared to be decreasing by day 5, but on day 7 the patient intended to resume AAS due to his depression, fatigue and cravings. |  |

**Table 2. Neuroendocrine (n=11)**

1. **Summary of condition**

| **#** | **Citation** | **Study design (country)** | **Patient(s) description** | **Patient(s) AAS status** | **Presentation setting and symptoms** | **Condition requiring treatment** |
| --- | --- | --- | --- | --- | --- | --- |
| 9 | van Breda et al., 2003 (Netherlands) | Case report | 37 year old male  Semi professional bodybuilder | 1 year history of AAS use with cessation a number of months prior to presentation. Had used a mixture of methylandrostenediol, stanozolol, nadrolone laurate and drostanolone propionate for 2x18 week cycles a year (details not reported). | Patient presented at exercise endocrinology unit with gynaecomastia and severe acne, headaches and weight gain. Symptoms persisted despite AAS cessation. | Hypogonadotropic hypogonadism. |
| 10 | Tan & Vasudevan, 2003  (USA) | Case report | 30 year old male  Bodybuilder | Used a mixture of nandrolone decanoate, deca Durabolin, primobolan depot and Winstrol (details not reported). | Presented with severe depression and loss of libido and energy. Had noticed testicular atrophy. | Hypogonadism. |
| 20 | Menon, 2003 (Malaysia) | Case report | Married couple: 27 year old woman and 37 year old man.  Male was an amateur bodybuilder. | Male patient had 10 year history of AAS use with cessation one year before referral. Cycles of use up to 8 months in length included testosterone cypionate (1g/week), dianabol (10-60mg/day), Anavar (20mg/day), Sustanon, Anadrol, Deca-Durabolin & Primobolan Depot (details not reported) | Referred by GP. Male patient had testicular atrophy and erectile dysfunction. | Complete azoospermia. |
| 33 | Gill, 1998 (UK) | Case report | 17 year old male  Competitive body builder | 6+ month history of AAS including a mixture of nandralone, Sustanon and stanazolol. | Presented with right testicular swelling and a varicocoele; erectile impotence for past 4 months, scant body hair, low libido, sore nipples. | Severe hypogonadotropic hypogonadism |
| 35 | Gazvani et al., 1997 (UK) | Case series | 4 males aged 28-37.  All cases reported as bodybuilders. | All current or recent AAS users. Details not reported. | Referred to andrology clinic with primary (n=3) or secondary (n=1) infertility | Azoospermia identified in all cases |
| 53 | Bickleman et al., 1995 (USA) | Case report | 29 year old male  Competitive bodybuilder | 8 month history of AAS use, discontinued one year prior to presentation. Used in alternating 16 week cycles of testosterone cypionate (1500-1800mg/week) and oxymetholone (560mg/week); followed by four week trial of hCG (dose not reported). | Sought endocrine consultation with impotence and low libido for one year since AAS cessation. | Impotence - reduced testicular volume on both sides and gynaecomastia on both sides. |
| 63 | Turek et al., 1995 (USA) | Case report | 34 year old male  Bodybuilder. | 5 year history of AAS use, discontinued 5 years prior to referral. Commonly used AAS in 14-16 week cycles followed by equal off cycle period. Most commonly used nandrolone decanoate and stanozolol. | Referred with azoospermia following treatment: Had been treated 2 years before this referral for low serum testosterone concentration with 2000 units HCG x3/week, followed by weekly doses of testosterone cyprionate (200mg), tapered over one year | Azoospermia |
| 64 | Cohen et al., 2005 (USA) | Retrospective chart review | 15 patients, mean age 33 years. | All patients had history of AAS use at time of presentation or previously. Mean time since AAS use was 5.3 years, with 5 patients actively using at time of presentation. | 9 patients presented at an infertility clinic. | Azoospermia |
| 67 | Priola et al., 2010 (Italy) | Case report | 34 year old male  Non-professional bodybuilder | 13 year history of AAS use, discontinued shortly prior to presentation. Cycles for the first ten years included nandrolone (25mg/day), stanazol (25mg/day) for 8 weeks; followed by mesterolone (50mg/day) for 15 days; followed by clomiphene citrate (50mg/day) for one week; followed by human chorionic gonadropin (3x 2000 IU/week) in one week. In the most recent 3 years the cycle also included boldenone (50mg/day for 3 weeks). | Presented to endocrinology department with loss of libido and energy, and mild depression. | Hypogonadotropic hypogonadism |
| 70 | Street and Scally, 2000 (USA) | Case series | 2 males aged 33 and 36 years old.  Both bodybuilders. | Case 1: 10+ year history of AAS use, total dose 500mg – 2g per week.  Case 2: 2 year history of continuous nandrolone use (200-400mg/week). | Both patients presented with hypogonadism. | Hypogonadism |
| 102 | Jarow and Lipshultz, 1990 (USA) | Case report | 36 year old competitive bodybuilder | 4 year history of AAS use for 6 months a year, discontinued 6 weeks prior to presentation. AAS included testosterone cypionate (200mg/week), nandrolone decanoate (200mg/week) and methandrostenolone (30mg/day). | Presented for evaluation for infertility as patient’s wife had not been able to conceive | Azoospermia |

1. **Treatment and outcomes**

| **#** | **Citation** | **Treatment provider and setting** | **Summary of treatment approach** | **Detail of medication(s) or therapy provided** | **Treatment outcomes** | **Comments** |
| --- | --- | --- | --- | --- | --- | --- |
| 9 | Van Breda et al., 2003 (Netherlands) | Not reported | Medical therapy | High dose LHRH injections on 3 consecutive days | Plasma testosterone levels remained within the normal range over the next 12 months indicating return to and maintenance of a normal hypothalamic-gonadotropic function |  |
| 10 | Tan & Vasudevan, 2003 (USA) | No reported | Medical therapy | Initially 100mg clomiphene citrate for 5 days successfully alleviated symptoms. Following relapse 2 months post-treatment clomiphene citrate treatment reinitiated for 2 months. | Symptoms resolved following the second treatment period. |  |
| 20 | Menon, 2003 (Malaysia) | Outpatient | Medical therapy | hCG injections 3x 10,000 IU/ week; hMG injections 75 IU/day for 3 months | Semen analysis and serum testosterone levels were normal; resolution of erectile difficulties; no impact on testicular volumes. Conception of baby 10 months after treatment began. |  |
| 33 | Gill, 1998 (UK) | Outpatient | Medical therapy | Received 250mg/ 2 weeks Sustanon for 15 months after refusing to discontinue AAS. Following discontinuing treatment and stopping AAS, patient given hCG injections to stimulate testicular function. Over 3 months he received 10,000 units/ week HCG for 1 month; 5000 units/ week HCG for one month; 2500 units/ week HCG for one month | Serum testosterone levels and potency returned to normal and remained normal after 30 months |  |
| 35 | Gazvani et al., 1997 (UK) | Outpatient | Advice to discontinue AAS | Not reported | Improvements in sperm concentration in all cases. Three cases confirmed pregnancy with partner during follow up times (up to 22 months). One case dropped out after 6 months and reinitiated AAS use. | Unclear if any treatment received beyond advice to stop AAS use, and no details on what this advice entailed. |
| 53 | Bickleman et al., 1995 (USA) | Outpatient | Medical therapy | Clomiphene therapy initially 50mg/day for one month, followed by 100mg/day for 3 weeks | Some improvement in first month, but when dose increased patient reported increases in libido and potency. Serum FSH, LH and testosterone levels returned to normal. Levels remained normal following therapy discontinuation. Patient returned to using AAS. |  |
| 63 | Turek et al., 1995 (USA) | Outpatient | Discontinuation of all medications; medical therapy | Chorionic gonadotropin (2000 IU x3/week for 4 weeks; followed by 3000 IU x3/week for 3 months). Received tamoxifen (10mg x2/day to minimize gynecomastia. | 3 months after therapy was initiated, patient’s wife conceived. Patient developed painful gynecomastia so human chorionic gonadotropin was discontinued and testosterone given intramuscularly |  |
| 64 | Cohen et al., 2005 (USA) | Outpatient | AAS cessation and medical therapy | Gonadotropin therapy | Azoospermia reversible in 78% cases. 5/9 patients required gonadotropin therapy for return of spermatogenesis and 2/9 patients had spontaneous return of spermatogenesis after cessation of AAS. Presumed irreversible in 2 patients. | Lacks detail – very short report |
| 67 | Priola et al., 2010 (Italy) | Outpatient | Medical therapy following AAS discontinuation | Triptorelin test 100ug x 1 administered | At ten days energy improved and testosterone levels improved to 7.0ng/mL. One month later serum testosterone was within normal range and patient reported normal libido and energy |  |
| 70 | Street and Scally, 2000 (USA) | Outpatients | Medical therapy | Both cases recevied 32 day therapy including hCG (2,500 IU every 4 days) clomiphene bid (50mg/QD) & tamoxifen (10mg/QD). For case 2 this was followed with 60 day therapy including hCG (4x5,000 IU every 4 days, followed by 4x2,500 IU every 4 days), clomiphene bid (50mg/QD), & tamoxifen (10mg/QD). This was followed with 32 day therapy including hCG (6x5000 IU QOD, followed by 6x2,5000 IU QOD) given simultaneously with menotropins (6x 150 IU QOD), clomiphene bid (50mg/QD) & tamoxifen (10mg/QD). | Testosterone levels increased following the first therapy for case 1 and the third therapy for case 2 |  |
| 102 | Jarow and Lipshultz, 1990 (USA) | Outpatient | Medical therapy | Injections of human chorionic gonadotropin | Following treatment, semen analysis demonstrated sperm in the ejaculate and the patient’s wife conceived 9 months later. | Paper includes a second case but no treatment described |

**Table 3. Hepatic (n=25)**

1. **Summary of condition**

| **#** | **Citation** | **Study design (country)** | **Patient(s) description** | **Patient(s) AAS status** | **Presentation setting and symptoms** | **Condition requiring treatment** |
| --- | --- | --- | --- | --- | --- | --- |
| 23 | Martin et al., 2008 (USA) | Case report | 27 year old male  Police officer and competitive bodybuilder | 5 year history of AAS use at first admission. Continued to use for 3.5 years after first treatment. Used androstenedione and nandrolone (details not reported) | Presented to A&E with midepigastric pain and nausea. Following treatment, returned 3.5 years later and presented with sudden-onset right upper quadrant pain. | Tumor haemorrhage in liver. On second presentation: tender hepatomegaly and haemorrhage, tachycardia. |
| 57 | Sanchez-Osorio et al., 2008 (Mexico) | Case report | 29 year old male | 3.5 month history of AAS use for prescribed by a personal trainer, including mesterolone (25mg/day), testosterone undecanoate (40 mg/day.), nandrolone undecanoate (30mg/ day), oxymetholone (50mg/day), stanozolol (10mg/day), testosterone (400mg/day). | Presented with 2 month history of abdominal pain and malaise followed by jaundice, choluria, oligocholia and intense pruritus; weight loss. | Liver toxicity, cholestasis |
| 71 | Chahla et al., 2014 (USA) | Case series | i) 36 year old male with history of hyperlipidemia  ii) 21 year old male athlete  iii) 42 year old male | All 3 patients had used over the counter supplements that had contained AAS (details not reported) | i) Presented with 8 week history of fatigue and pruritis, anorexia and weight lost, dark coloured urine and jaundice  ii) Presented with worsening abdominal pain and malaise, nausea and vomiting for 6 weeks.  iii) Presented with 2 week history of worsening jaundice, malaise and pruritus. | Hepatoxicity identified in all 3 cases. |
| 72 | Awai et al., 2014 (USA) | Case report | 18 year old male with a history of non-alcoholic steatohepatitis, obesity and hypertension | 2 weeks use of Beastrdrol (40mg/day) prior to admission | Presented with 3 days of scleral icterus, intermittent emesis and difficulty concentrating and staying awake | Liver toxicity |
| 73 | Krishnan and Gordon, 2009 (USA) | Case series | i) 21 year old male  ii) 30 year old male  iii) 38 year old male | In all cases, patients had used supplements that contained AAS | Patients presented with symptoms including: jaundice, worsening pruritis, fatigue, nausea and weight loss and scleral icterus | In all cases hepatotoxicity was identified. In case iii) patient suffered from renal failure. |
| 74 | Socas et al., 2005 (Spain) | Case series | i) 35 year old male bodybuilder  ii) 23 year old male bodybuilder | i) 15 year history of AAS including most frequently stanozolol, oxymetholone, nandrolone decanoate, testosterone enanthate and methenolone enanthate at high doses (400mg daily for oral AAS, 600mg 2-3 times a week injectable AAS) in cycles of 8 weeks on and 2 weeks off.  ii) 6 month history of AAS use including stanozolol, oxymetholone, nandrolone decanoate, testosterone phenylpropionate and boldenone in cycles of 8 weeks on and 2 weeks off. | i) Patient was included in an experimental examination programme for bodybuilders and was asymptomatic.  ii) admitted to emergency department in a confused state. | i) hyperechogenic lesions in the liver.  ii) acute renal failure, muscular damage, metabolic alkalosis and hypernatraemia. |
| 79 | Patil et al., 2007 (UK) | Case report | 43 year old male  Bodybuilder | 25 year history of AAS up to 4 years prior to presentation. Had used nandrolone decanoate, stanozolol, primoabolain, and various forms of testosterone (details not reported, but suggests doses were relatively low). | Presented in emergency department after collapsing at home. Reported epigastric pain for 2 days. | Hepatic rupture with cardiovascular collapse, sepsis and acute renal failure |
| 81 | Solbach et al., 2015 (Germany) | Case report | 29 year old male  Professional bodybuilder | 1 year history of AAS use in cycles of 4 weeks with rest periods, stopped 5 weeks before presentation. Used nandrolone decanoate (400mg/week), sustanon (750mg/week), methandienone (280-350mg/week), stanozolol (50mg/day for 1 month) and human growth hormone (4 IE/day for 3 months). Also used diuretics before competition, insulin and tamoxifen. | Presented at emergency department with midepigastric pain | Hepatocellular carcinoma. Liver was enormously enlarged. |
| 84 | Gorayski et al., 2007 (Australia) | Case report | 35 year old male bodybuilder | Recent AAS use over 2 months and previous 10 year history of use. Recent use included testosterone (200mg QID) and stanozolol (50mg/day). | Presented with 2 day history of chest pain, cough, breathlessness and fever | Hepatocellular carcinoma |
| 85 | El Khoury et al., 2017 (Lebanon) | Case report | 35 year old male | 12 week history of AAS use and dietary supplements. Used stanozolol (100mg x3 times/week), trenbolone (100mg x3 times/week) & testosterone propionate (50mg x3 times/week). | Reported yellow skin discolouration 5 weeks prior to presentation; presented to hospital with jaundice, colicky abdominal pain, vomiting and severe pruritus. | Liver injury resulting in prolonged cholestasis and acute kidney injury. |
| 93 | Li et al., 2018 (China) | Case report | 22 year old male  Bodybuilder | Used AAS for 10 days two weeks priors to admission including stanozolol (10mg/day), clenbuterol (40ug/day) for 7 days, then doubled the dose for 3 days. Used triiodothyronine (25 ug/day) for all 10 days. | Presented to hospital with symptoms of progressive dyspnoea, paroxysmal nocturnal dyspnoea, intermittent cough and bloody sputum | Dilated cardiomyopathy and acute hepatic injury. |
| 94 | Stepien et al., 2015 (Poland) | Case report | 19 year old male  Amateur bodybuilder | 2 month history of stanozolol use (50mg/every other day) | Admitted to hospital with general malaise, jaundice and severe itching | Severe intrahepatic cholestasis that developed to severe liver failure |
| 101 | Elsharkawy et al. 2012 (UK) | Case series | i)32 year old male  ii)16 year old male | i) used methandrostenolone (5mg/day) for previous 2 months  ii) used methandrostenolone for five days (details not reported) before onset of symptoms 3 weeks previous to admission. | i)presented with 7 day history of nausea, vomiting, jaundice and severe itching  ii)presented with 3 week history of severe itching, nausea and abdominal discomfort | Cholestasis in both cases. Case 1 became severely hypertensive and case two had a mild element of hepatitis. |
| 109 | Ampuero et al., 2014 (Spain) | Case report | 37 year old male | Used AAS for previous 3 week. Had used stanozolol at high doses. | Admitted with severe jaundice and pruritus | Severe cholestasic jaundice |
| 110 | Singh et al., 2008 (USA) | Case series | Three males aged 51, 25 and 33 years | All cases had used methasteron alongside other supplements | All cases presented with jaundice and pruritus | Servere hepatoxicity, cholestasis |
| 111 | Boks et al., 2017 (Netherlands) | Case report | 24 year old male | 2 months prior to presentation patient had completed 8 week cycle of trenbolone enanthate (50mg/day); 1 year previously completed 8 week cycle of testosterone (250ml/week). | Presented with loss of appetite, dark coloured stool, yellow stool and yellow sclerae. Developed pruritus. | Mild jaundice; cholestatic hepatitis identified through liver biopsy |
| 113 | Winwood et al., 1990 (UK) | Case report | 30 year old male  Amateur powerlifter | 18 month AAS history including stanozolol (15mg/day), methandienone (15mg/day), oxandrolone (7.5mg/day) | Admitted having vomited blood. Later vomited 1500 ml blood and clots. | 3 grade II oesophageal varices |
| 133 | Ding et al., 2013 (Australia) | Case report | 46 year old male | 2 month history of AAS, stopping 5 months before onset of symptoms including stanozolol (40mg/day) and methandrostenolone (40mg/day). | Presented to emergency department with 3 month history of jaundice and weight loss of 9kg over 3 weeks | Intrahepatic cholestasis |
| 135 | Nasr & Ahmad, 2009 | Case report | 42 year old male | Used AAS for 3 weeks prior to onset of symptoms. Used methasteron (100 tablets Superdrol). | Presented with 4 week history of jaundice, pruritus, dark urine and weight loss | Severe cholestasis and renal failure. Re-admitted with pruritus a week after discharge. |
| 140 | Marcacuzco Quinto et al., 2014 (Spain) | Case report | 30 year old male  Bodybuilder | States that patient had a long-term history of synthetic steroid and hormone use (details not reported) | Presented to emergency department with asthenia, abdominal pain, nausea and vomiting. | Hepatic rupture, liver failure. Hematoma of the liver with active bleeding and hemoperitoneum. |
| 141 | Hymel et al., 2013 (USA) | Case report | 26 year old male | Used Mastabol for 10 days prior to onset of symptoms (details not reported). | Presented with 3 week history of jaundice, dark coloured urine, light coloured stools, pruritus and weight loss. | Acute, nonobstructive, intrahepatic cholestatic hepatitis |
| 143 | Flores et al., 2016 (USA) | Case report | 31 year old male | 16 week history of AAS use prior to admission including cumulative daily dose of 104mg AAS as part of a herbal and dietary supplements regimen that included 80+ ingredients. | Reported fatigue and jaundice, developed pruritus, weight loss and hallucinations during treatment. | Severe jaundice. Patient was readmitted for concerns of encephalopathy and liver failure. Bile acid nephropathy identified. |
| 146 | Diaz et al., 2016 (Spain) | Case series | Four males aged 20-29 years | All cases had used epitiostanol (21-58 days) starting 30-33 days before onset of symptoms | All cases presented with jaundice and pruritus. Two patients reported asthenia | Severe cholestasis, hepatoxtoxicity |
| 152 | Pais-Costa et al., 2012 (Brazil) | Case report | 28 year old male  Bodybuilder | 6 year history of AAS including androstenedione and nandrolone (details not reported). | Referred with abdominal pain the right upper quadrant for past 3 months | Giant hepatic adenoma |
| 165 | Hardt et al., 2012 (Germany) | Case report | 37 year old male  Professional bodybuilder | Five year history of AAS use including a wide range of substances, as well as daily intake of amino acids, vitamins and growth hormones. | Presented to hepatology department with increasing epigastric and abdominal pain | Hepatocellular carcinoma |

1. **Treatment and outcomes**

| **#** | **Citation** | **Treatment provider and setting** | **Summary of treatment approach** | **Detail of medication(s) or therapy provided** | **Treatment outcomes** | **Comments** |
| --- | --- | --- | --- | --- | --- | --- |
| 23 | Martin et al., 2008 (USA) | Inpatient | Surgery; instruction to stop using AAS | Initially underwent left lateral hepatic segmentectomy with open cholecystectomy. Instructed to discontinue AAS. Upon readmission patient underwent angiographic embolization of the right hepatic artery, red blood cell transfusion | Discharged after 5 days post operation |  |
| 57 | Sanchez-Osorio et al., 2008 (Mexico) | Not reported | Medical therapy; AAS discontinued | Ursodeoxycholic acid treatment (15mg/kg). | Symptoms and cholestasis persisted for 2 months, but by 5 months patient was recovering well |  |
| 71 | Chahla et al., 2014 (USA) | Mixed | Medical therapy | i) patient treated with cholestyramine and diphenhydramine for symptom control  ii) treated with hydroxyzine and ursodeoxycholic acid on discharge  iii) patient treated conservatively and symptomatically with questran, hydroxyzine, ursodeoxycholic acid, analgesics and topical creams for pruritus. | Recovery over 10-12 weeks in all cases |  |
| 72 | Awai et al., 2014 (USA) | Outpatient | Medical therapy | Therapy initiated with ursodeoxycholic acid and lactulose. After one month, Cholestyramine added to the regimen. Hydroxyzine and rifampin prescribed to treat severe pruritus. Eight days after, direct bilirubin increased to 20.9 and liver biopsy performed which demonstrated cholestatic hepatitis suggestive of immunoallergic drug-induced hepatotoxicity. | Symptoms abated after 3 months. | Patient had prior liver problem |
| 73 | Krishnan and Gordon, 2009 (USA) | Mixed | Medical therapy | i) started on oral prednisone (40mg/day with slow taper).  ii) started on hydroxyzine and cholestyamine for symptom relief  iii) discharged on oral hydroxyzine and ursodiol for 3 weeks, and then started on oral prednisone (40mg/day with slow taper). | In all three cases, over 1-3 months the patients’ condition improved and normalised | Not that clear on treatments or diagnosis in all cases |
| 74 | Socas et al., 2005 (Spain) | i) outpatient  ii) inpatient in nephrology unit | i) instruction to discontinue AAS; inclusion in liver transplanatation program; ii) patient received haemodialysis; instruction to discontinue AAS. | ii) patient received 3 haemodialysis sessions | i) 4 years after diagnosis, tumors had slightly decreased in size and liver function had improved. Patient remained in transplantation program and did not use AAS.  ii) Discharge once renal failure, asthenia and confusion were resolved. At 1 year patient was recovering well. |  |
| 79 | Patil et al., 2007 (UK) | Inpatient | Resuscitation, surgery | Required aggressive resuscitation with blood products and fluids for cardiovascular collapse and underwent emergency laparotomy and abdomen was packed. | Patient made an uncomplicated but slow recovery and was discharged to ward after 20 days |  |
| 81 | Solbach et al., 2015 (Germany) | Inpatient | Chemoembolization was declined by patient who was recommended for transplantation | Transplantation | Transplantation was performed without complication. 27 months after, patient was in excellent condition and not using AAS. |  |
| 84 | Gorayski et al., 2007 (Australia) | Inpatient | Surgery | Patient underwent a right hemi-hepatectomy. | Not reported | Outcome of treatment not reported. Bit unclear about the details. |
| 85 | El Khoury et al., 2017 (Lebanon) |  | Patient advised to stop using AAS, medical therapy, plasma exchange. Patient refused renal biopsy. | Initially intravenous fluids given including sodium bicarbonate to prevent cast formation, albumin, ursodeoxycholic acid and hydroxyzine for pruritus. A total of 20,200ml plasma volume used over 6 sessions during plasma exchange. | Patient tolerated plasma exchange and was discharged home. At three weeks, patient displayed clinical and laboratory improvement and was completely asymptomatic at 3 and 9 month follow up. |  |
| 93 | Li et al., 2018 (China) | Inpatient | Medical therapy | Described as symptomatic and supportive medical therapy | Cardiac markers and liver function test showed gradual improvement and patient was discharged after 12 days. |  |
| 94 | Stepien et al., 2015 (Poland) | Inpatient | Medical therapy | Initial conservative treatment included L-ornithine L-aspartate, timonacic and ursodexycholic acid and other therapy (not reported). After 3 weeks, steroid therapy including hydrocortisone initiated (200mg/ day for 2 days then at gradually reduced dose for 10 days). | Patient was discharged after 2 months. Five months after discontinuing stanozolol use all hepatic enzymes had returned to normal values. | Unclear on some details of treatment |
| 101 | Elsharkawy et al. 2012 (UK) |  | Medical therapy | i) initially treated with ursodeoxycholic acid. In response to becoming hypertensive he was treated with amlodipine, and with chlorphenamine and colestyramine for itching.  ii) suffered from severe refractory pruritus and treated with colestyramine, chlorphenamine, ursodeoxycholic acid, rifampicin and naltrexone over 15 days. | i) bilirubin fell from day 4 and patient was discharged on day 7. Liver function returned to normal over 4 months.  ii) Patient had prolonged admission. Suffered from anxiety following discharge but liver function returned to normal over 4 months. |  |
| 109 | Ampuero et al., 2014 (Spain) | Inpatient | Unclear | States patient was provided with ‘supportive medical treatments’ | At 8 weeks patient was completely asymptomatic and had discontinued stanozolol | Lacks detail on treatment |
| 110 | Singh et al., 2008 | Inpatients | Medical therapy | Pruritus treated across patients with medications including cholestyramine, ursodeoxycholic acid, hydroxyzine, doxepin, diphenhydramine therapy and rifampicin. In one case plasmaphereses was required after medications failed. | In all cases, liver injury was resolved in 2-5 months after stopping Superdrol (Methasterone) use. Pruritus was successfully treated in all cases. |  |
| 111 | Boks et al., 2017 (Netherlands) |  | Medical therapy. | Pruritus treated with cholestyramine and later rifampicin | At 2 months, pruritus diminished and patient was recovering | Lacks detail on treatment |
| 113 | Winwood et al., 1990 (UK) | Inpatient | Blood transfusion and sclerotherapy | Blood transfusion of two units acutely | At two weeks patient was recovering and remained well during the next 6 months. Discontinued AAS. |  |
| 133 | Ding et al., 2013 (Australia) | Inpatient | Medical therapy | Received ursodeoxycholic acid treatment for 7 weeks | At 7 weeks, patient was discharged with complete resolution of jaundice and treatment was ceased |  |
| 135 | Nasr & Ahmad, 2009 | Inpatient and outpatient | Medical therapy. | Discharged after 4 days with prescribed oral ursodeoxycholic acid (600mg/x2 day) and hydroxyzine (25mg as required) for pruritus. On re-admission he was discharged on naltrexone for pruritus. | After one month renal function had normalised but patient remained jaundiced. At 6 weeks pruritus had significantly improved with normalisation kidney function. After 4 months bilirubin normalised. |  |
| 140 | Marcacuzco Quinto et al., 2014 (Spain) | Inpatient | Surgery | Right hepatic artery was ligated and perihepatic packing was used, and the abdomen was left open with a Bogota bag that was removed after 48 hours, and necrotic liver segment was resected. After 72 hours, necrosis was identified which was resected and the laparotomy closed. | Hepatic and renal functions successfully corrected. | Lacks detail on outcomes |
| 141 | Hymel et al., 2013 (USA) |  | Medical therapy | Patient placed on phenobarbital for pruritus. | States that clinical symptoms resolved | Unclear if other treatment received or follow up length |
| 143 | Flores et al., 2016 (USA) | Inpatient | Medical therapy, blood transfusion | Patient was started on IV solumedrol (30mg/day) for 11 days, but jaundice did not improve. On re-admittance patient received 5 sessions of plasmapheresis, and blood transfusion. | Patient was discharged on day 20 after re-admittance. Six weeks later he was asymptomatic and recovering well. |  |
| 146 | Diaz et al., 2016 (Spain) | Inpatient | Medical therapy followed by MARS therapy | Sates that patients received standard medical treatment for 1 month with no improvements. Following this, patient received 7 hour sessions of MARS per day for 2-3 consecutive days. | Following MARS treatment, pruritus disappeared, renal function and other markers improved, and patients were discharged. At follow up 4 months later, all patients were recovering well. |  |
| 152 | Pais-Costa et al., 2012 (Brazil) | Inpatient in ICU | Surgery | Bilateral subcostal incision with median upper prolongation performed. Right hepatectomy by means of an anterior approach undertaken and simple enucleation was performed to treat the lesion. | Patient was kept in ICU for one day and discharged on day 8. He stopped using AAS and symptoms and abnormalities were not present at six month follow up. |  |
| 165 | Hardt et al., 2012 (Germany) | Inpatient | Surgery | Laparoscopic segmentectomy of the liver performed and tumour was resected | Patient was discharged on day 7 after uneventful clinical course. At 27 month follow up, there was no sign of recurrence. |  |

**Table 4. Kidney disorders (n=6)**

1. **Summary of condition**

| **#** | **Citation** | **Study design (country)** | **Patient(s) description** | **Patient(s) AAS status** | **Presentation setting and symptoms** | **Condition requiring treatment** |
| --- | --- | --- | --- | --- | --- | --- |
| 75 | Kesler et al. 2014 (USA) | Case report | 24 year old male  Competitive bodybuilder | 7 year history of AAS (testosterone 200mg/week) at time of initial presentation (2004). Upon re-admission (2008), patient stated he did not use AAS. | Presented initially with acute abdominal pain(2004). Re-admitted at 4 and 9 years. | Initial diagnosis of hepatic adenomatosis (2004). On third admission, diagnosed with chronic kidney disease and coronary artery disease (2013). |
| 76 | Merino Garcia et al., 2018 (Spain) | Case report | 37 year old male with history of hypertension  Bodybuilder | Used testosterone and stanozolol, as well as growth hormone and creatine (details not reported) | Presented at emergency department with signs of malaise, nausea, headache and blurred vision for one week. | Severe acute kidney failure with high blood pressure, anaemia and thrombocytopenia |
| 91 | Tarashande et al., 2018 (Iran) | Case report | 33 year old male with history of substance misuse including methadone, opium and tramadol. | Used oxymetholone (details not reported) to prevent weight loss from other substance use | Presented at emergency department with decreased urine volume, urine colour change, and lower abdominal pain. | Acute renal failure; muscle injury and rhabdomyolysis |
| 139 | Daher et al., 2009 (Brazil) | Case series | i)21 year old male athlete  ii)30 year old make | i)1 month history of AAS and veterinary supplements (50ml/week).  ii)2 year history of AAS use as well as vitamins and dexamethasone supplements (total 12 mg every 2 weeks). | i)Presented to emergency department with abdominal pain, nausea and vomiting. One month prior to admission experienced abdominal pain, nausea, vomiting, dizziness and weakness, headache, fever and profuse sweating.  ii)Presented to emergency department with persistent vomiting | Acute kidney injury in both cases |
| 153 | Colburn et al., 2017 (USA) | Case report | 43 year old male with prior appendectomy | 5 year history of testosterone and trenbolone acetate up to 2 weeks priors to admission (details not reported) | Presented to emergency department with 2 days left flank pain. Repeated representations with pain following discharge | Recurrent renal infarction |
| 160 | Samaha et al., 2008 (Lebanon) | Case report | 25 year old male | 2 month history of AAS use (details not reported) alongside diuretics and amino acid supplements. | Presented at emergency room with abdominal pain, nausea and vomiting | Acute pancreatitis, acute renal failure and hypercalcemia. |

1. **Treatment and outcomes**

| **#** | **Citation** | **Treatment provider and setting** | **Summary of treatment approach** | **Detail of medication(s) or therapy provided** | **Treatment outcomes** | **Comments** |
| --- | --- | --- | --- | --- | --- | --- |
| 75 | Kesler et al. 2014 (USA) | Outpatient | Advice given to discontinue AAS, but patient continued to use. Surgery undertaken in response to chronic kidney disease and coronary artery disease in 2013. | In 2013 required haemodialysis and placement of 7 coronary artery stents. Patient was given trans-arterial embolization with micron LC Beads mixed with 50mg Doxorubicin and iodinated contrast. | Patient suffered complications and poor health after initial admission, which led to subsequent admissions |  |
| 76 | Merino Garcia et al., 2018 (Spain) | Inpatient in ICU | Medical therapy | Acute haemodialysis and plasmapheresis started simultaneously. Administered IV nitrates and labetalol and oral treatment with an ACE inhibitor. | Hypotensive medication and plasmapheresis discontinued after 4 sessions. Further outcomes not reported | Limited outcomes reported |
| 91 | Tarashande et al., 2018 (Iran) | Inpatient in department of nephrology | Medical therapy | 7 rounds of haemodialysis and hydration | Eight days after admission CPK and LDH levels declined rapidly and the patient was discharged with ARF was settled and values normalised |  |
| 139 | Daher et al., 2009 (Brazil) | Inpatient | Medical therapy. | Hydration with saline 0.9% and furosemide initiated to treat hypercalcemia. Prednisone (1mg/kg/day) initiated on day 7. On discharge, patient continued maintenance therapy with oral prednisone for 6 weeks.  ii) Venous hydration and furosemide administered, as well as prednisone (1mg/kg/day). | i) patient became stable and was discharged with recovering renal function after 20 days.  ii)patient became stable after 1 month and was recovering renal function at discharge. |  |
| 153 | Colburn et al., 2017 (USA) | Outpatient and inpatient | Medical therapy, counselling | Range of therapies including opioid analgesics, heparin drip, apixaban, and therapeutic INR on warfarin. States that extensive counselling provided regarding the risks of continued AAS use. | Patient was discharged after final presentation following stabilisation and pain control. |  |
| 160 | Samaha et al., 2008 (Lebanon) | Inpatient | Medical therapy | Treatment included aggressive fluid therapy, furosemide, proton pump inhibitors and symptomatic treatment, and pethidine injections for pain | After 10 days patient was recovering and was discharged |  |

**Table 5. Cardiovascular (n=26)**

1. **Summary of condition**

| **#** | **Citation** | **Study design (country)** | **Patient(s) description** | | **Patient(s) AAS status** | **Presentation setting and symptoms** | **Condition requiring treatment** |
| --- | --- | --- | --- | --- | --- | --- | --- |
| 5 | Shimada et al., 2012 (Japan) | Case report | 27 year old male | | 6 month history of methasterone and prostanozol use (details not reported) | Patient admitted to hospital due to right hemiparalysis, homonymous hemianopia, dysarthria, tinnitus and double vision that occurred during muscle training | Cardioembolic stroke |
| 14 | Sveinsson & Herrman 2013  (Sweden) | Case report | 21 year old male  Bodybuilder | | 4 month history of dianabol use (20mg/day) | Admitted with paraesthesia and dyspraxia in left hand followed by generalised tonic-clonic seizure. Symptoms included drowsiness, disorientation and sensory inattention for left arm, pronation drift of the left arm and positive extensor response on left side. | Blood clot: Intraparenchymal haemorrhage in right parietal lobe; right cortical venous thrombosis |
| 56 | Garg et al., 2010 (UK) | Case report | 24 year old male | | Used AAS (details not reported) | Presented to hospital with acute chest pain and borderline anterior ST elevation | Acute myocardial infarction. |
| 58 | Shamloul et al., 2014 (Egypt) | Case report | 37 year old male | | 2 year history of AAS use including most frequently methandienone and methenolone acetate (details not reported) | Presented to neurocritical care unit with acute right sided weakness, confusional state following a first generalised tonic clonic seizure. | Severe toxic cardiomyopathy. |
| 60 | Luc et al., 2018 (Canada) | Case report | 26 year old male with history of substance use including alcohol, cocaine and methamphetamine. | | 3 year history of testosterone, trenbolone and primobolan use (details not reported) and human growth hormone. | Presented to A&E with shortness of breath, hemoptysis and presumed diagnosis of worsening pneumonia. | Cardiomyopathy |
| 78 | Santamarina et al., 2008 (Argentina) | Case report | 26 year old male  Amateur athlete | | 3 month history of stanozolol use (10mg/day) for previous 3 months | Presented in emergency department with headache, vomiting and loss of consciousness | Posterior territory ischemic stroke. |
| 86 | Edvardsson 2014 (Sweden) | Case report | | 20 year old male  Bodybuilder | 3 month history of methandrostenolone use (25 mg/day). | Presented with severe headache followed by nausea, vomiting, visual disturbances, confusion and sleepiness. | Severe hypertension, posterior reversible encephalopathy syndrome |
| 88 | Sonmez et al., 2016 (Turkey) | Case report | 32 year old male  Bodybulder | | Three year history of methenolone acetate use (200mg/week) | Presented at emergency department with severe chest pain that had spread to neck and arm | Acute coronary syndrome |
| 90 | Falkenberg et al. 1997 (Sweden) | Case series | i)A 37 year old male athlete and bodybuilder; ii)27 year old male bodybulder | | i) 10 year intermittent history of AAS use (details not reported); ii) 13 months history of AAS use during 3 periods with increasing doses. In most recent 2 month cycle, used methandione (50 mg/day), stanozolol (30mg/day), oxymetholone (50 mg/day). | i) sudden onset of pain in left leg  ii) Sudden onset of pain in right calf and foot | i) occlusion of all major arteries of the leg.2 years later patient was admitted to clinic: no pulse could be felt in left groin.  ii) Arterial thrombosis: occlusion of the upper and middle thirds of all arteries in lower leg |
| 104 | Laroche, 1990 (Canada) | Case report | 28 year old male  Competitive bodybuilder | | 3 year history of AAS use including twice monthly injections of stanozolol, oxandrolone, nandrolone decanoate, trembolone acetate, chorionic gonadotropins and methyltestoterone | Admitted to hospital with complaints of speech difficulties and loss of sensitivity on right side; some memory loss. Had become weak, dizzy and unable to talk during a wrestling match 3 days earlier. | Stroke. Upon readmission 3 years later, identified severly ischemic right lower limb caused by diffused distal arterial thrombosis |
| 108 | Youssef et al., 2011 (Kuwait) | Case report | 39 year old male  Bodybuilder | | 3 year history of nandrolone use (no details reported). | Presented with dizziness and expressive aphasia for previous 6 hours. Three months earlier pateitn presented with transient ischaemic attack in sudden loss of vision in left eye and weakness in left upper and lower limbs. He had refused admission to hospital and was discharged on aspirin. | Cardiomyopathy, stroke and peripheral vascular disease |
| 122 | Bispo et al., 2009 | Case report | 40 year old male  Bodybuilder | | 10 year history of AAS use over in cycles of 6-10 weeks with 2-3 week off cycle periods. Stopped one month prior to admission. Most frequently used AAS were methandrostenolone, stanozolol, oxymetholone, nandrolone decanoate, testosterone enanthate and trenbolone enanthate. Reported very large doses. | One month prior to admission patient had experienced fatigue, decreased exercise tolerance and general malaise. Symptoms continued and he developed anorexia, vomiting, abdominal pain and jaudince. | Severe toxic cardiomyopathy. |
| 123 | Goldstein et al., 1998 (USA) | Case report | 26 year old male  Competitive bodybuilder | | 3 year history of AAS including intermittent use of testosterone propionaite, cypionate and enanthate; methandrostenolone and stanozolol. Had not used AAS for 4 weeks prior to presentation and in that time had used clenbuterol. | Presented at outpatient clinic with dull, central chest pain of 3 hours’ duration. | Myocardial infarction |
| 124 | Gunes et al., 2004 (Turkey) | Case report | 43 year old male | | Used AAS including drostandon propionate, testosterone propionate and methandrostenolone (no details reported) | Presented to emergency department with chest pain. | Myocardial infarction |
| 126 | Christou et al., 2016 (Greece) | Case report | 30 year old male | | Used AAS for 2 months prior to admission including stanozolol (10mg/day) and twice a week testosterone propionate (30mg), felinpropinat testosterone (60mg), testosterone izocaproat (60mg), testosterone decanoate (100mg). | Admitted following acute episode of severe substernal pain and diaphoresis | Myocardial infarction |
| 128 | Stergiopoulos et al., 2008 (USA) | Case report | 44 year old male  Recreational weight lifter | | 2 year history of intermittent AAS including the previous 6 weeks. Used testosterone as well as sildenafil occasionally (details not reported) | Presented with one day of intermittent exertional, substernal chest pain; left shoulder pain, shortness of breath and diaphoresis. | Acute myocardial infarction and polycythaemia |
| 129 | Santos et al., 2015 (Portugal) | Case report | 25 year old male  Practiced bodybuilding | | 6 month history of AAS use with previous cycle initiated 6 weeks prior to admission that included oxandrolone (40mg/day), clenbuterol (0.08mg/day), mesterolone (50mg/day), hCG (10 IU/day), nandrolone (600mg/ 2xweek), stanozolol (100mg/ 3x week), drostanolone (200mg/ 3x week), trenbolone (200mg/ 3x week), testosterone propionate (100mg/ 3x week), boldenone (400mg/ 2x week) & methenolone (200mg/ 2x week). | Presented in emergency care following intense opporessive retrosternal pain that lasted for 2 hours and was associated with muscle fatigue after training. 24 laters the pain recurred and worsened. | Myocardial infarction |
| 130 | Yihan et al., 2010 (Turkey) | Case report | 41 year old male  Bodybuilder | | 15 year history of oxymetholone and methenolone use (details not reported) | Admitted to emergency department with chest pain | Acute inferior myocardial infarction. During treatment, diagnosed with large renal infarction in the right kidney. |
| 131 | Huie, 1994 (USA) | Case report | 25 year old male | | 16 week history of nandrolone decanoate use (100mg/week for 6 weeks, 4 week off cycle, 200mg/week for 6 weeks) up to 2 weeks prior to admission | Presented at emergency department with severe crushing substernal chest pain | Acute myocardial infarction |
| 132 | Ferenchick & Adelman, 1992 (USA) | Case report | 37 year old male  Competitive weight lifter | | 7 year history of AAS use with current 16 week cycle including nandrolone decanoate (200mg/wk), Oxandrolone (50mg/day) and Boldenone, Testosterone cypionate, stanozolol veterinary (doses unknown) | Presented with 45-minute history of chest pain radiating to left arm and nausea, diaphoresis and shortness of breath. Pain began during squat lifts. | Myocardial infarction |
| 137 | Lau et al., 2007 (Australia) | Case report | 36 year old male  Bodybuilder | | 12 week history of AAS use, which coincided with onset of symptoms. Used testosterone ethanate (250mg-1.5g/ week), Stanozolol (50-200mg/ week). | History over 6 months of irregular palpitations, significant fatigue without syncope, dyspnea or chest pain. | Persistent atrial fibrillation |
| 138 | Mewis et al., 1996 (Germany) | Case report | 28 year old male | | 2 year history of stanozolol (280mg/week) use discontinued 6 months prior to admission. | Referred with Ventricular tachycardia with heart rate of 150 beats/min. | Severe coronary heart disease |
| 142 | Ment & Ludman, 2002 (UK) | Case report | 23 year old male  Bodybuilder | | 3 month history of methandrostenelone use (20mg/day) | Presented with recent onset of chest pain | Coronary thrombus |
| 145 | Ahlgrim & Guglin, 2009 (USA) | Case report | 41 year old male  Bodybuilder, history of cardiomyopathy and continued heavy weightlifting exercise after cardiac failure. | | 1 year history of AAS use before first admission including 2 regimens of testosterone enanthate (250mg/ every 5 days x 6 weeks) and weight lost drugs. Authors believe doses may be understated and patient also used insulin like growth factor 1. | Presented initially to emergency department with abdominal tenderness and 2 week history of nausea. One year later admitted with breathlessness at rest, fatigue and paroxysmal nocturnal dyspnoea. 2 weeks prior to this, a dobutamine pump had been installed. | Cardiomyopathy; severe systolic dysfunction and Class IV heart failure. |
| 149 | Joseph et al., 2017 (USA) | Case report | 33 year old male | | One year history of AAS use (details not reported) | Referred with progressive lower extremity oedema, dyspnoea and orthopnoea with onset 2 weeks prior to admission. | Cardiomyopathy; acute systolic heart failure. |
| 164 | Nieminen et al., 1996 (Finland) | Case series | 4 cases aged 27-33 years | | All patients had history of AAS use from 2 to 8 years including intermittent and more continuous use | One patient attended for a check up because of his history of AAS use. Three patients were referred with ventricular fibrillation during exercise, clinically manifest heart failure and arterial thrombosis respectively. | Cardiac hypertrophy. In two cases there were symptoms of heart failure including in one, massive thrombosis in both ventricles. |

1. **Treatment and outcomes**

| **#** | **Citation** | **Treatment provider and setting** | **Summary of treatment approach** | **Detail of medication(s) or therapy provided** | **Treatment outcomes** | **Comments** |
| --- | --- | --- | --- | --- | --- | --- |
| 5 | Shimada et al., 2012 (Japan) | Hospital inpatient | Medical therapy, AAS use discontinued | Aspirin therapy | No recurrence of cerebral infarction | Unclear on what, if anything, the treatment entailed beyond aspirin treatment and discontinuing AAS |
| 14 | Sveinsson & Herrman 2013 (Sweden) | Hospital inpatient | Anticoagulation therapy | Anticoagulation therapy with intravenous heparin for 11 days and oral anticoagulation (warfarin) for 4 months | Full recovery reported after 4 months and treatment ceased |  |
| 56 | Garg et al., 2010 (UK) | Inpatient | Surgery | Treated initially with intravenous abciximab bolus followed by 12 hour infusion. After one week, patient received further treatment with abciximab. | Treatment noted as successful |  |
| 58 | Shamloul et al., 2014 (Egypt) | Inpatient | Medical therapy | Slow isotonic saline infusion initiated to correct hyponatraemia. In ICU, therapy with cardiac inotropes and intensive diuresis initiated. Low-molecular weight heparin therapy initiated followed by oral anticoagulation. | Patient discharged from ICU following improvement but later developed further symptoms. Following transfer back to ICU patient died. |  |
| 60 | Luc et al., 2018 (Canada) | Inpatient | Incubation, medical therapy, resuscitation, dialysis and device implantation, addiction counselling referral | Patient required intubation for respiratory failure, as well as multiple vasopressors and inotropes for cardiogenic shock. Suffered from multiple asystolic cardiac arrests requiring cardiopulmonary resuscitation and acute renal failure requiring PRISMA initiation. Patient received temporary circulatory support converted to a durable HeartMate II LVAD device. He was referred to addictions counselling. | Patient made gradual and steady improvement and was discharged after 34 days post operation. 18 months later there were indications of recovery, which continued following discontinuation of LVAD device. States he was compliant with counselling. |  |
| 78 | Santamarina et al., 2008 (Argentina) | Inpatient | Intubation and ventilation; rehabilitation | Patient was intubated and ventilated for 15 days with nasogastric tube feeding. Following extubation he started rehabilitation in hospital | After 45 days patient was discharged with severe disability. He had right hemiparesis, spasticity and hyperreflexia, limitation of the ocular movements, mixed aphasia, and severe dysarthria. |  |
| 86 | Edvardsson 2014 (Sweden) | Inpatient | Aggressive treatment of blood pressure with intravenous drugs; AAS use stopped | (Not reported) | Patient was discharged after 5 weeks. At 3 weeks, regressions in abnormalities and symptoms were absent at 6 and 12 months |  |
| 88 | Sonmez et al., 2016 (Turkey) | Outpatient | Medical therapy | Prescribed oral diltiazem (60 mg) three times a day and Ramipril (2.5 mg) once a day | At one week follow up patient displayed no symptoms |  |
| 90 | Falkenberg et al., 1997 (Sweden) | Outpatients | i) surgery  ii) Initially thrombolysis attempted with no improvement. Surgery performed. | i) 8mm PTFE graft from the common iliac to the deep femoral artery was used in combination with a femorotibial bypass using an in situ saphenous vein graft.  ii) Bypass from the popliteal artery to the most distal portion of the posterior tibial artery performed | i) patient was discharged free of symptoms after 1 week. Re-admitted 18 months later due to gradual return of pain, re-operation took place. One year later patient had severe cramping pain and walking difficulty, but no rest pain.  ii) patient developed progressive gangrene of the distal foot leading to a fore-foot amputation. Lab studies at 3 month follow up revealed only minor abnormalities or normal results. |  |
| 104 | Laroche, 1990 (Canada) | Inpatient | Medical therapy | Treated with acetylsalicylic acid for 2 days (325mg bid) as inpatient and as outpatient for 2 months (325mg bid). On re-admission, treated with ASA, heparin and dipyridamole for 6 days. Following discharge, treated with ASA and dipyridamole. | At 6 months after re-admission patient was recovering well. He had developed gynaecomastia. 3 years later patient was reported to remain well and involved in discouraging AAS use amongst others | States that patient discontinued all AAS following second discharge only, and did not reinitiate |
| 108 | Yousseff et al., 2011 (Kuwait) | Inpatient | Medical therapy | Managed with intravenous unfractionated heparin infusion, statins, angiotensin converting enqume inhibitors and beta blockers. Patient was discharged on aspirin and warfarin. | At 3 month follow up, resolution of thrombus with partial improvement of ejection fraction demonstrated. At 6 months, ankle brachial index was improved. |  |
| 122 | Bispo et al., 2009 | Inpatient | Medical therapy | Hyponatremia corrected with saline. Therapy included dopamine, dobutamine and watchful diuresis. Four days post-admission, weight heparin therapy started. | Patient discharged after 16 days and was recovering well with improved ventricular function, disappearance of intraventricular thrombus and no lesions identified. |  |
| 123 | Goldstein et al., 1998 (USA) | Inpatient | Medical therapy | Received aspirin and nitroglycerin. | States that patient had an uneventful hospital course. Two weeks after discharge he was asymptomatic and recovering well. |  |
| 124 | Gunes et al., 2004 (Turkey) | Not reported | Medical therapy | Treated with Clopidogrel (300mg and 75mg/day thereafter), and Tirofiban infusion administered for 48 hours. Statin and angiotensin converting enzyme inhibitor added to treatment to correct cholesterol levels. | After 3 weeks patient performed well at treadmill test without pain |  |
| 126 | Christou et al., 2016 (Greece) | Inpatient | Medical therapy | Conservative treatment included aspirin (100mg/day), tricagrelor (90mg x2/day), bisoprolol x7.5mg/day), Ramipril (5mg/day), rosuvastatin (10mg/day), pantoprazole (40mg/day) for 6 days | Patient was described as having an uneventful hospital course but refused to stay after 6 days and did not undergo any further medical examination. | Notes that patient was a heavy smoker |
| 128 | Stergiopoulos et al., 2008 (USA) | Inpatient | Surgery, medical therapy, phlebotomy. | Percutaneous intervention of the right coronary artery. Therapy included aspirin, clopidogrel, beta-blocker, statin and angiotensin converting enzyme inhibitor. Patient was phlebotomised until resultant haematocrit was 45%. | Intervention and phlebotomy were successful | Notes history of smoking and family history of early coronary artery disorder. Limited outcomes reported. |
| 129 | Santos et al., 2015 (Portugal) | Inpatient | Medical therapy. | Patient was started on double platelet antiaggregant therapy with acetylsalicylic acid and clopidogrel and anticoagulation therapy with fondaparinux.  Patient discharged with aspirin (150mg od), clopidogrel (75 mg od), bisoprolol (5mg od), Ramipril (2.5mg od) and rosuvastatin (10mg od). | Patient responded well and remained electrically and hemodynamically stable without pain. He was discharged on day 8 with medication prescription. At one year follow up he was asymptomatic and abstinent from AAS. |  |
| 130 | Yihan et al., 2010 (Turkey) | Inpatient | Medical therapy, surgery | Initially administered 300mg aspirin and 600mg clopidogrel. Received 10,000 IU intravenous heparin followed by primary percutaneous coronary intervention.  To treat renal infarction, enoxaparain (1mg/kg x 2/day) added to aspirin and clopidogrel treatment. Prescribed antiplatelet therapy and anti-ischemic on discharge. | Discharged on 10^th^ day. Examination indicated patient was recovering well including complete revascularisation, stent patency and improved blood flow. |  |
| 131 | Huie, 1994 (USA) | Inpatient | Medical therapy, rehabilitation | Patient was given tissue plasminogen activator and started on aspirin and intravenous metoprolol, nitroglycerin and heparin. He received intracoronary urokinase infusion to restore coronary blood flow and aggressive diuresis, inotropic therapy with dobutamine and captopril in response to cardiogenic pulmonary oedema. Following discharge on aspirin, metoprolol, captopril and benzonatate perles for cough after 13 days, he began a 12 week cardiac rehabilitation programme. | Patient completed rehabilitation. At 8 months patient had not used AAS and had no cardiac symptoms. |  |
| 132 | Ferenchick & Adelman, 1992 (USA) | Inpatient | Medical therapy | Treated with intravenous tissue plasminogen activator and heparin | Reports that patient recovered uneventfully | Patient took up to 20 aspirin a day for headaches |
| 137 | Lau et al., 2007 (Australia) | Outpatient | Electrical cardioversion, medical therapy. | Elective direct current cardioversion performed and patient commenced on warfarin. When atrial fibrillation occurred again about 15 days, patient was commenced on flecainide (100mg x 2/day), metropolol (150mg x 2/day). Patient also stopped using AAS at this point. Second direct current cardioversion attempted 4 months later – 300j cardioversion failed and 360 J cardioversion restored normal heart rate. | Patient remained with a normal heart rate for over 1 year after cessation of all therapy. Patient ceased AAS use during treatment after the first cardioversion. |  |
| 138 | Mewis et al., 1996 (Germany) | Inpatient | Surgery, medical therapy | Received 50mg Ajmaline to restore normal heart rate. Percutaneous transluminal coronary angioplasty of the right coronary artery performed and patient discharged with 200mg amiodarone and antianginal therapy including aspirin. | Tachycardia successfully treated and angioplasty was successful. No follow up reported. |  |
| 142 | Ment & Ludman, 2002 (UK) | Unclear | Medical therapy | Received abciximab, aspirin and low molecular weight heparin. | 48 hours after provision of treatment, complete dissolution of all thrombus. States that patient made an uneventful recovery. |  |
| 145 | Ahlgrim & Guglin, 2009 (USA) | Inpatient | Medical therapy and device implementation until discharge. On re-presentation, medical therapy. | Aggressive diuresis with furosemide initiated. Dobutamine pump removed following improvement and patient discharged with external defibrillator. Following readmission, given continuous milrinone (0.25mg/kg/min). | Patient improved following re-admission but states that evaluation for heart transplant was initiated. |  |
| 149 | Joseph et al., 2017 (USA) | Inpatient | Medical therapy. Patient was instructed not to use AAS at discharge. | Patient stabilised on norepinephrine and milrinone drips that were ultimately weaned off. Patient was transitioned to oral heart failure regimen including metoprolol and enalapril. | At time of discharge patient had mild symptoms and could walk without difficulty. He was advised not to use AAS. At 10 month follow up with continuation of standard medical therapy for heart failure he remained asymptomatic and had not used AAS. |  |
| 164 | Nieminen et al., 1996 (Finland) | Inpatient | Surgery, medical therapy. In 1 case no treatment was reported. | In cases 2-4: ii) programmed electrophysiological stimulation performed, isoprenaline infusion; iii) right sided catheterisation performed; iv) embolectomy performed | Notes that two patients stopped using AAS. Recovery in three patients noted. One was lost to follow up. |  |

**Table 6. Musculoskeletal (n=13)**

1. **Summary of condition**

| **#** | **Citation** | **Study design (country)** | **Patient(s) description** | **Patient(s) AAS status** | **Presentation setting and symptoms** | **Condition requiring treatment** |
| --- | --- | --- | --- | --- | --- | --- |
| 4 | Stannard & Bucknell, 1993 (USA) | Case report | 35 year old male  Soldier and physical trainer in the army. Formerly a competitive bodybuilder. | 5 year history of AAS use, discontinued 6 months prior to injury. Use included testosterone cypionate (200mg daily), nandrolone decanoate (100mg weekly), oxymetholone (50mg daily) used for 3-4 x12 week cycles per year. | Unclear setting.  Presented with injured left elbow that occurred while lifting weights.  Patient had previously had elbow injury for 18 months treated with six steroid injections over a 1 year period. | Tear in the mid-substance of the triceps tendon. |
| 38 | Farkash et al., 2009 (Israel) | Case report | 39 year old male  Amateur bodybuilder | 7 year history of AAS use (details not reported) | Presented in A&E with severe pain and inability to move right shoulder following AAS injection and excessive exercise that day | Massive rhabdomyolysis |
| 105 | Fenelon et al., 2016 (Ireland) | Case report | 29 year old male  Amateur weight lifter | Current AAS user (details not reported) | Severe pain and swelling in lower anterior thighs, onset was during squat in competition. | Quadriceps tendon rupture, patella tendon rupture, distal femur fracture, patella dislocation in both legs |
| 106 | Bagherifard et al., 2018 (Iran) | Case report | 32 year old male  Body builder | Recent history of AAS including nandrolone decanoate (200mg/week), testosterone depot (500mg/week), trenbolone acetate (200mg/2x a week), boldenone (200mg/3x a week) for 12 weeks; followed by 500 iu HCG x 10 days, then Nolvadex (40mg/day x 6 weeks). This was followed by another cycle, and continued for 6 months after injury. | Referred to emergency department with pain, swelling and ecchymosis in knees since falling down stairs 2 weeks earlier. Previous fall 10 months ago led to an ecchymosis and bulging around superomedial aspect of right elbow. | Quadriceps tendon rupture in both knees and partial rupture of triceps tendon. |
| 114 | Liow & Tavares, 1995 (UK) | Case report | 29 year old male  Competitive body builder | Had used AAS up to 11 months previously  Had used combination of stanozolol, nandrolone decanoate, methandrostenolone and clembutarol (doses and cycle not reported) | Presented with pain and swelling in both knees and inability to stand following a jump from height of 15 feet. Had pain in the quadriceps tendon prior to injury | Bilateral rupture of the quadriceps tendon |
| 115 | Kramhoft & Solgaard, 1986 (Denmark) | Case report | 42 year old male  Bodybuilder | 4 month history of AAS  including Nandrolone decanoat (200mg300mg/ week) with two months use of Ethyl-estreonl followed by two months Nandrolone fenylpropionat for two months (details not reported). | Admitted due to lack of extension of the distal phalanx of the left thumb following leg-exercises, preceded by pain in the left wrist over previous two months | Complete rupture of the extensor pollicis longus tendon. |
| 116 | Tapaninen et al., 2016 (Finland) | Case report | 51 year old male | Current AAS user  Had used AAS for 10 years including testosterone and nandrolone (doses and cycles not reported). | Patient experienced sudden severe pain both knees after squat exercises, was unable to stand or walk or raise a straight leg. | Complete bilateral quadriceps tendon rupture identified in both legs |
| 117 | David et al., 1995 (UK) | Case report | 32 year old male  Professional bodybuilder | 10 year + history of AAS.  Details not reported but states very high doses. | Experienced sudden severe pain in both knees while attempting squat lift | Rupture of both quadriceps tendons |
| 118 | Visuri & Lindholm, 1994 (Finland) | Case report | 23 year old male  Army conscript, competitive bodybuilder. | 6 year history of AAS including weekly intake of 1500-2000mg of AAS (e.g. oxandrolone, oxymethelone, stanozolol, methandostenolone, methelone enanthate, testosterone enanthate), increased before competitions. Also used 1500 IU/week HCG and tamoxifen (10mg/day). In the past year, intake was reduced. | Patient admitted to hospital having felt snap in left elbow during boxing training and biceps muscle pulled upwards and was painful. Had previous detached the distal head of the right biceps tendon that had been surgically reattached. | Bilateral distal biceps tendon avulsions |
| 134 | Freeman & Rooker, 1995 (UK) | Case report | 22 year old male  Bodybuilder | 8 weeks history of oxymethalone (200mg/day) prior to symptom onset. | Patient had stumbled and his knee had given way several times since | Complete rupture of the anterior cruciate ligament |
| 144 | Adamson et al., 2004 (UK) | Case report | 25 year old male | Had used stanazolol ‘several times’ and metenolon on one occasion, including recent use (details not reported). | Presented with bilateral thigh and calf pain that occurred during cardiovascular workout. Was sent home on diclofenac tablets but re-presented 2 days later with difficulty walking | Rhabdomyolysis. Initially diagnosed with musculoskeletal pain. |
| 161 | Erturan et al., 2013 (UK) | Case report | 25 year old male  Bodybuilder | AAS user (no details provided) | Presented to emergency department following road traffic accident, pain developing 2 hours later. | Bilateral simultaneous traumatic upper arm compartment syndromes |
| 167 | Leopardi et al., 2006 (Italy) | Case report | 28 year old male  Bodybuilder | 3 year history of AAS use including, per year, stanozolol (500mg/week), metanolone enanthate (500mg/week) and growth hormone (8IU/day) for 3 months, followed by nandrolone (800mg/week), testosterone (500mg/week), T4 (100mg/day). Cycle repeated twice per year. | Presented to emergency department following road traffic accident with weakness, knee pain and inability to extend right knee. Patient was referred 5 months later, following immobilisation and physical therapy with lack of extension. | Complete tear of quadriceps tendon |

1. **Treatment and outcomes**

| **#** | **Citation** | **Treatment provider and setting** | **Summary of treatment approach** | **Detail of medication(s) or therapy provided** | **Treatment outcomes** | **Comments** |
| --- | --- | --- | --- | --- | --- | --- |
| 4 | Stannard & Bucknell, 1993 (USA) | Hospital inpatient | Surgery, immobilisation | Following surgery the patient was started on active range of motion with a brace that limited flexion and extension after 3 weeks. Range of motion gradually increased for 3 months before cast was removed. | Nine months post operation the patient was performing full duties as a fitness trainer and able to lift weights. Range of motion at 18 months was greater on the injured side. | Noted that cause of injury may have been either personal AAS use, steroid injections (cortisol) or combination |
| 38 | Farkash et al., 2009 (Israel) | Inpatient | Medical therapy | Intravenous fluid replacement and sodium bicarbonate to alkalise the urine | Pain was reduced after 4 days, full recovery followed |  |
| 105 | Fenelon et al., 2016 (Ireland) | Inpatient and then outpatient during recovery | Surgery, immobilisation, physiotherapy | Operative repair with both legs immobilised in a fixed extension brace for 6 weeks following  surgery. | Operative repair was successful. Follow-up at 5 months revealed an extension lag of five degrees. right knee laxity was noted and ACL tear diagnosed and treated with standard ACL rehabilitation and physiotherapy |  |
| 106 | Bagherifard et al., 2018 (Iran) | Inpatient and then outpatient during recovery | Surgery, immobilisation, physiotherapy | Quadriceps tendon repared through surgery followed by immobilisation for 6 weeks and physiotherapy, which was continued during recovery | Six months later patient was recovering well. AAS was discontinued. | Bit unclear on current/ recent AAS use. Triceps damage treatment not reported. |
| 114 | Liow & Tavares, 1995 (UK) | Inpatient and then outpatient during recovery | Surgery, immobilisation | Tendons repaired with strong sutures passed through drill-holes in the patellae. Both legs immobilised for 8 weeks. | Four months after surgery, the patient was recovering well – could straight leg raise with minimal lag and could flex up to 60 degrees. |  |
| 115 | Kramhoft & Solgaard, 1986 (Denmark) | Outpatient | Surgery, immobilisation | End to end repair of the tendon carried out through surgery, and plaster cast with hyperextension of the thumb applied for 6 weeks | 3 months after surgery, the patient had regained full function of the thumb and normal strength. Patient had stopped using AAS. |  |
| 116 | Tapaninen et al., 2016 (Finland) | Outpatient | Surgery, immobilisation | Tendons reattached with sutures using three vertical bone tunnels to patella. Retinacula  were also repaired. Following surgery, both legs were immobilised for 8 weeks and range of motion gradually increased. | Three months after operations, patient as able to walk normally and maintain full weight bearing; able to make straight leg raise with minimal lag and could flex knees up to 90 degrees. After 1 year he could flex knees up to 115-120 degrees. |  |
| 117 | David et al., 1995 (UK) | Outpatient | Surgery, immobilisation, physiotherapy | Repair performed on each side followed by immobilisation in casts for 6 weeks. Physiotherapy initiated. | 9 months after operation the left leg still had an extension lag of 15 degrees. Patient had difficulty rising from chair and used sticks to walk. At 12 months, extensor lag was 5-10 degrees and patient no longer needed crutches to walk. |  |
| 118 | Visuri & Lindholm, 1994 (Finland) | Outpatient, military hospital | Surgery, immobilisation, physiotherapy | On both occasions, the tendons were reattached using the Boyd-Anderson method. After 5 week immobilisation of the elbow in 90 degree of flexion, active physiotherapy was started. | 9 weeks after both operations patient achieved full range of motion in the elbow joints. |  |
| 134 | Freeman & Rooker, 1995 (UK) | Outpatient | Physiotherapy | Sates that patient was treated conservatively with physiotherapy | At one year follow up patient reported that his knee occasionally gave way, but did not wish to progress to ligament repair. Patient had not used AAS in that time. |  |
| 144 | Adamson et al., 2004 (UK) | Unclear | Medical therapy | Initially treated with intravenous fluids, diazepam and paracetamol; hydration and urinary alkalinisation. Following muscle biopsy patient was started on oral prednisolone followed with methylprednisolone. | Mobility improved and patient recovered during treatment, and was discharged. |  |
| 161 | Erturan et al., 2013 (UK) | Inpatient | Surgery | Bilateral fasciotomies of both anterior and posterior compartments were performed and both humeri underwent open reduction and internal plate fixation. | At long term follow up the patient made good recovery and fractures healed without complications |  |
| 167 | Leopardi et al., 2006 (Italy) | Inpatient | Surgery, immobilisation, rehabilitation | Reconstruction of the quadriceps tear using ipsilateral hamstring tendons followed by 6 weeks immobilisation. Patient then initiated 4 months of rehabilitation. | Patient began using AAS following rehabilitation. At 37 months follow up the patient had full passive motion, walked normally and was competing again. |  |

**Table 7. Infectious complications (n=7)**

1. **Summary of condition**

| **#** | **Citation** | **Study design (country)** | **Patient(s) description** | **Patient(s) AAS status** | **Presentation setting and symptoms** | **Condition requiring treatment** |
| --- | --- | --- | --- | --- | --- | --- |
| 103 | Rich et al., 1999 | Case report | 26 year old male  Bodybuilder | AAS user (no details provided). Reported sharing vials with 2 other users who both developed abscesses. | Patient presented to GP with pain in thigh at injection site and later at emergency room following treatment, again with pain | Abscess. Patient had soft-tissue infection at injection site with pain, erythema and heat 2 weeks after presentation. |
| 156 | Friendman et al., 2016 (Israel) | Case report | 35 year old male  Amateur bodybuilder | 4 year history of AAS including recent 3 week use of Trenbolone. | Referred by GP following no improvement following prescribed medication for injection site pain and secretion | Full thickness skin and subcutaneous tissue necrosis |
| 162 | Shiber et al., 2013 (USA) | Case report | 45 year old male  Amateur bodybuilder | Recent AAS use (no details provided) | Presented with right thigh pain and swelling for 3 days following injection 1 week prior | Pyomyositis |
| 163 | Evans, 1997 (UK) | Case series | 30 and 28 year males  Competitive and recreational bodybuilders | Both patients had recent AAS use, including i)stanozolol and ii)nandrolone decanoate | One patient presented with a painful and swollen right knee and one with short history of paraesthesia over the dorsum his left hand. | Injection injury. In both cases, patients had injected near to the complication. |
| 166 | Tuzel, 2015 (Turkey) | Case report | 38 year old male | 10 year history of nandrolone decanoate and stanozolol use (details not reported) | Presented with 1 week history of penile swelling and pain | Spontaneous corpus cavernosum abscess |
| 168 | Grant, et al., 2010 (UK) | Case report | 25 year old male bodybuilder | AAS history not reported, but had injected AAS six days prior to admission | Admitted in septic shock with limitation of shoulder movement with non-fluctuant swelling from acromio-clavicular joint to postero-lateral and anterior deltoid. | Necrotizing myositis |
| 170 | Marquis & Maffulli, 2006 (UK) | Case report | 36 year old male bodybuilder | Patient reported 10 day AAS cycle including 100mg testosterone propionate, 300mg testosterone enathate, 300mg nandrolone (history not reported) | Presented with bilateral deltoid abscesses (4cmx5cm) associated with pyrexia and marked local erythema | Abscess |

1. **Treatment and outcomes**

| **#** | **Citation** | **Treatment provider and setting** | **Summary of treatment approach** | **Detail of medication(s) or therapy provided** | **Treatment outcomes** | **Comments** |
| --- | --- | --- | --- | --- | --- | --- |
| 103 | Rich et al., 1999 (USA) | Outpatient | Medical therapy; counselling | Initially patient prescribed oral cephalexin for 1 week; and then clarithromycin for 2 weeks followed by one injection of ceftriaxone. Ciprofloxacin added to clarithromycin for 1 week. At 6 weeks post-presentation, 200cc gross pus drained and GP inserted a Penrose drain. Following presentation at emergency room 2 weeks later, patient treated with cefazolin 500mg IV every 6 hours. Received counselling on risks of hepatitis B and HIV and side effects from steroid use. | 1 week after discharge, patient’s wound was healing well. Did not return for further follow up. |  |
| 156 | Friendman et al., 2016 (Israel) | Outpatient | Surgery | Underwent surgical wound debridement to remove necrotic tissue and promote healing and treatment of wound | Patient discharged with clean wounds which healed well by 3 weeks follow up. Complete epithelialization observed at 2 months. |  |
| 162 | Shiber et al., 2013 (US) | Inpatient | Medical therapy, surgery | Received intravenous vancomycin and piperacillin-tazobactam. Incision and washout during surgery. | Patient recovered uneventfully and was discharged with antibiotics after 5 days. |  |
| 163 | Evans, 1997 (UK) | Inpatient then Outpatient | Surgery, medical therapy | In one case the patient’s knee was lavaged arthroscopically and he was given antibiotics. In the second case, the injury recovered without treatment | In both cases, patient’s recovered. |  |
| 166 | Tuzel, 2015 (Turkey) | Inpatient | Surgery | Tunica albuginea was opened longitudinally and 10ml pus was drained. Placement of a small penrose drain and injection of broad-spectrum antibiotics for 1 week. | Symptoms disappeared after surgery. At 3 month follow up patient was recovering well, but for mild left-sided penile deviation on erection |  |
| 168 | Grant, et al., 2010 (UK) | Inpatient then outpatient | Surgery, medical therapy | Patient underwent emergency drainage with debridgement of large areas of necrotic muscle and a fasciotomy of the upper arm. Treated with benzyl penicillin and clindamycin. | Retained excellent shoulder function despite extensive muscle debridgement |  |
| 170 | Marquis & Maffulli, 2006 (UK) | Inpatient then outpatient | Surgery, medical therapy | Both abscesses were incised and drained; the wounds were packed. Antibiotics commenced with flucloxacillin & benzyl penicillin, followed by restricted to flucloxacillin only | Follow up over 2 months with wounds healing well at discharge. Patient intended to continue AAS use. |  |

**Table 8. Other (n=8)**

1. **Summary of condition**

| **#** | **Citation** | **Study design (country)** | **Patient(s) description** | | **Patient(s) AAS status** | **Presentation setting and symptoms** | **Condition requiring treatment** |
| --- | --- | --- | --- | --- | --- | --- | --- |
| 6 | Ray et al. 2008 (UK) | Case report | 47 year old male  Professional bodybuilder | | 2-3 year history of AAS use including Deca-Durabolin (300mg/week); Testoviron (200mg/week) and Sustanon 250 (300mg/week). | Patient presented at otolaryngology department as an emergency with breathing difficulties and voice changes over 4-5 months prior to admission | Chronic laryngitis associated with generalised supraglottic and glottis swelling with restriction on the airway. |
| 83 | Maini et al., 2014 (UK) | Case report | 30 year old male  Hoping to improve physique and muscle mass. History of cocaine and alcohol use, but not at time of admission. | | Used 50g of stanozolol in week prior to admission | Presented at emergency department after a witnessed seizure, patient was incoherent. | Hypokalaemia and metabolic alkalosis |
| 87 | Labib and Haddon 1996 (UK) | Case report | 29 year old male  Bodybuilder | | Current AAS user with weekly doses of testosterone cypionate (200mg/ml) and nandrolone decanoate (200mg/mL) and intermittent courses of fluoxymesterone and testosterone undecanoate. | Referred to lipid clinic with abnormal lipid profile | Abnormal lipid profile |
| 89 | Cooper et al., 2011 (UK) | Case report | 46 year old male  Recreational bodybuilder | | 20 year history of AAS use including nandrolone used cyclically with varying doses and intermittent use of testosterone and human growth hormone. | Admitted originally with lower limb cellulitis and 6 days later with abdominal pain and sepsis.  Following treatment was re-admitted to ICU after collapsing. | Acute respiratory distress syndrome identified after collapse. |
| 136 | Unai et al., 2013 (USA) | Case report | 42 year old male  Amateur bodybuilder | | Used AAS (states for a few years) including testosterone acetate, testosterone cypionate, decanoate, propionate, pheylpropionate, enathate and isocaproate. | Presented to emergency department with nausea, vomiting, diarrhoea and 5 days of shortness of breath and cough. | Patient was diagnosed with multiple organ dysfunction syndrome, acute kidney injury and refractory supraventricular tachycardia |
| 155 | Geraci et al., 2017 (USA) | Case report | 33 year old male | 3.5 month history of AAS use that discontinued 3 weeks before presentation. Use included two types of testosterone and trenbolone acetate. | | Presented to emergency department with range of symptoms including polydipsia, polyuria, nausea, blurry vision, headache, malaise and hyperglycaemia. | New onset of diabetes |
| 158 | Alaraj et al., 2004 (Lebanon) | Case series | 32 and 24 year old males, weight lifters | Patients had 7 and 1 year histories of AAS and growth hormone use (details not reported) | | Both patients presented to emergency department with headaches. Case 1 also had symptoms including visual blurring, nausea and vomiting. | Spontaneous subdural haematoma |
| 159 | Moor et al., 2005 (UK) | Case report | 36 year old male  Professional bodybuilder | | History of AAS use (details not reported) and recent use of growth hormone at both 2 months and 1 week prior to presentation. | Presented with increasing hoarseness and breathing difficulties, particularly during sleep | Bilateral internal laryngoceles |

1. **Treatment and outcomes**

| **#** | **Citation** | **Treatment provider and setting** | **Summary of treatment approach** | **Detail of medication(s) or therapy provided** | **Treatment outcomes** | **Comments** |
| --- | --- | --- | --- | --- | --- | --- |
| 6 | Ray et al. 2008 (UK) | Hospital inpatient | Medical therapy followed by laser treatments | Upon admission: three times daily intravenous dexamethasone (8mg), cefuroxime (750g), metronidazole (500mg).  Following elective tracheostomy the patient was treated with three laser resections followed by decannulation | Improvements in voice, laryngeal airway and hypertrophic laryngitis following laser treatments. Persistent hypertrophy in vestibular folds remained. | Notes that patient was also a regular smoker for 20 years. |
| 83 | Maini et al., 2014 (UK) | Inpatient | Fluid provision | Parenteral fluid rehydration and potassium replacement | Over 48 hours the electrolyte imbalances and alkalosis were corrected and conscious level returned to normal. Patient was discharged but did not attend follow up appointments. |  |
| 87 | Labib and Haddon 1996 (UK) | Outpatient at lipid clinic | Advice to stop using AAS | Advised to stop exercising and using non-prescribed drugs | Six weeks later patient had lost 8.2kg weight and changes in serum lipid results were addressed. | No treatment beyond advice to stop exercising and using AAS |
| 89 | Cooper et al., 2011 (UK) | Inpatient in Stroke Unit. | Intubation and ventilation; rehabilitation. | Patient ventilated for 12 hours and provided with inotrope support for 2 days. During rehabilitation on then stroke unit, patient encountered complications including pulmonary embolism requiring lifelong warfarin management, and pancreatic, managed conservatively. | Patient was discharged home with residual right upper limb weakness and dyspraxia, requiring daily assistance from carers. | Not all treatment clearly described |
| 136 | Unai et al., 2013 (USA) | Inpatient | Resuscitation, medical therapy, ventilation, haemodialysis and electrical cardioversion for different symptoms. | Initially, patient was volume resuscitated and pharmacologic anti-arrhythmic therapy initiated, with medical therapy initiated for preliminary diagnosis of pneumonia. Following development of hypoxia patient was placed on continuous mandatory ventilation until day 27, and VV-ECMO initiated and placed on a regimen of piperacillin/tazobactam (3.375g every 8 hours) and moxifloxacin (400mg/ x4 daily). Veno-veno haemodialysis initiated for fluid removal. Atrial flutter attempted to be resolved through diltiazem/ amiodarone protocol and electrical cardioversion and adenosine provision. Patient eventually placed on oral amiodarone and diltiazem. | Patient came off ventilator on day 27 and nausea and vomiting resolved gradually. Tracheostomy removed prior to discharge on day 38. | Notes previous history of smoking |
| 155 | Geraci et al., 2017 (USA) | Inpatient | Medical therapy, advice | Intially received intravenous fluids, a bolus of 10 units regular insulin and an insulin drip, and serum potassium supplements. Discharged with education how to manage blood glucose, signposting to GP and advice not to use AAS or growth hormones. | Patient stabilised over first 24 hours and was discharged |  |
| 158 | Alaraj et al., 2004 (Lebanon) | Inpatient | Surgery | Both patients underwent burr hole evacuation of the haematoma. | In both cases, surgery was successful and headaches subsided |  |
| 159 | Moor et al., 2005 (UK) | Inpatient | Medical therapy | Initially treated with intravenous corticosteroids and broad-spectrum antibiotics which led to improvement. Deterioration following discharge led to re-admission and further corticosteroid treatment. | Patient responded well to treatment and was discharged. Symptoms recurred seven months later, but patient has since remained stable |  |
