# Supplementary material for: Treatments for people who use anabolic androgenic steroids: a scoping review
Source: Harm Reduct J. 2019 Dec 30;16:75. doi: 10.1186/s12954-019-0343-1 (PMC6937954; doi:10.1186/s12954-019-0343-1)
Supplement: Supplementary file 2 — Additional file 2. Search strategy. The full search strategy used in Medline is provided. [file 12954_2019_343_MOESM2_ESM.docx]

**Search strategy**

**Search strategy for Medline**

The database search was developed in Medline and adapted for the other databases searched.

|  | ***Concept*** | **Terms for searching within titles and abstracts** |
| --- | --- | --- |
| 1. | *Steroid terms* | (anabolic N2 steroid*) or doping or ((performance N2 enhanc*) or (image N2 enhanc*) N3 (drug* or substance*)) |
| 2 |  | (MH “Anabolic Agents”) OR (MH “Performance-Enhancing Substances”) OR (MH “Doping in Sports”) |
| 3 |  | 1 OR 2 |
| 4 | *Health impact/ condition terms* | dependen* or addict* or withdraw* or (adverse or negative or undesir* or side or harm* (N3 acute or chronic or health or effect or impact)) or injur* or (blood borne virus*) or BBV or (infectious N2 disease*) |
| 5 |  | (MH "Hepatitis C") OR (MH "Hepatitis B") OR (MH “HIV”) OR (MH "Drug-Related Side Effects and Adverse Reactions") OR (MH “Substance-Related Disorders”) OR (MH “Substance Withdrawal Syndrome”) OR (MH “Endocrinology”) OR (MH “Cardiovascular Diseases”) OR (MH “Liver Diseases”) OR (MH “Musculoskeletal Diseases”) OR (MH “Dermatology”) OR MH “Mood disorders” OR MH “Depression” |
| 6 |  | 4 OR 5 |
| 7 | *Intervention terms* | treat* or therap* or intervention* or cessation or support or care or procedur* or medicat* or medicine or (needle (N2 exchange or program*)) or pharmacy or (drug or substance) N2 (practitioner* or service or center or centre)) or doctor or physician* or (general N1 practic*) |
| 8 | *Search for articles that include terms for all three searches* | 3 AND 6 AND 7 (HUMAN) |
